# Genomic and Experimental Analysis of the Biostimulant and Antagonistic Properties of Phytopathogens of *Bacillus safensis* and *Bacillus siamensis*

**DOI:** 10.3390/microorganisms10040670

**Published:** 2022-03-22

**Authors:** Fabiola Altimira, Sebastián Godoy, Matias Arias-Aravena, Bárbara Araya, Christian Montes, Jean Franco Castro, Elena Dardón, Edgar Montenegro, Wilson Pineda, Ignacio Viteri, Eduardo Tapia

**Affiliations:** 1 Laboratorio de Entomología y Biotecnología, Instituto de Investigaciones Agropecuarias, INIA La Platina, Santiago 8831314, Chile; fabiola.altimira@inia.cl (F.A.); godoy.gonzalez.s@gmail.com (S.G.); matias.arias@ug.uchile.cl (M.A.-A.); b.arayavega@gmail.com (B.A.); 2 Department of Plant Pathology and Microbiology, Iowa State University, Ames, IA 50011, USA; chrisfm@iastate.edu; 3 Banco de Recursos Genéticos Microbianos, Instituto de Investigaciones Agropecuarias, INIA Quilamapu, Chillán 8831314, Chile; jean.castro@inia.cl; 4 Centro de Excelencia Microbiano, El Jocotillo 01065, Guatemala; elena.dardon@popoyan.com.gt (E.D.); edgar.montenegro@popoyan.com.gt (E.M.); wilson.pineda@popoyan.com.gt (W.P.); iviteri@popoyan.com (I.V.)

**Keywords:** *Bacillus safensis*, *Bacillus siamensis*, biostimulant, antifungal activity, *Botrytis cinerea*, *Colletotrichum acutatum*, *Fusarium oxysporum*, *Phytophthora cinnamomi*

## Abstract

The *B. safensis* RGM 2450 and *B. siamensis* RGM 2529 strains were isolated from the rhizosphere of plants presenting resilience to abiotic and biotic stress conditions. To understand the implications of bacteria in resilience, a genomic and experimental analysis was carried out on their biostimulant and phytopathogenic antagonist properties. Genome analyses of both strains indicated that they have the potential to synthesize bioactive compounds such as the battery of non-ribosomal peptides, polyketides, extracellular enzymes and phytohormones. These results were consistent with the antagonistic activities of both strains against the phytopathogens *Botrytis cinerea*, *Colletotrichum acutatum*, *Fusarium oxysporum* and *Phytophtora cinnamomi*. They also showed the capacity to solubilize phosphorus, fix nitrogen and produce indole acetic acid. This was observed in tomato seedlings grown from seeds inoculated with the mixture of strains which presented significantly greater length as well as wet and dry weight in comparison with the treatments individually inoculated with each strain and the control. Accordingly, the combination of *B. safensis* RGM 2450 and *B. siamensis* RGM 2529 showed synergistic biostimulant activity. These findings contribute new knowledge of the genomic and metabolomic properties taking part in the symbiotic interactions between these strains and the plants and uphold the combined use of both strains as a biostimulant.

## 1. Introduction

Plant-growth-promoting rhizobacteria (PGPR) are soil bacteria which colonize plant rhizospheres and increase their growth, constituting a powerful tool in agriculture and degraded soil restoration [1,2,3]. The positive effects mediated by PGPR have been described in a wide range of plants in various environmental conditions, including important commercial crops such as wheat, corn, rice and tomatoes. The last of these has a production of around 180 million tons annually, representing one of the most important plant crops today [4]. Both the growth and yield of tomatoes are reported to be promoted by PGPR strains such as *Pseudomonas fluorescens* 63-28, *Bacillus fortis* IAGS162, *Bacillus subtilis* IAGS174,13 *Burkholderia tropica* MTo-29314 and *Gluconacetobacter diazotrophicus* 15 [5].

Among PGPR, there is great scientific and economic interest in the *Bacillus* genus since it is genetically diverse and widely distributed in various ecological niches. It presents the capacity to form spores, which allows it to be a stable bioinoculant in the soil [6]. Between 5% and 10% of its genome is involved in biosynthesis of antimicrobial molecules [7,8]. These molecules can include ribosomal peptides (RPs), non-ribosomal peptides (NRPs) and polyketides (PKs). RPs were originally called “bacteriocins”, since they inhibit the growth of bacteria closely related to the producing strain. Bacteriocins’ action modes include protoplasm vesicularization, pore formation and cell disintegration [9,10].

NRPs are synthesized by non-ribosomal peptide synthases (NRPS), which catalyze the formation of peptide links between different amino acid substrates due to their proteinic organization in the form of biosynthetic modules. Each module is responsible for incorporating a particular amino acid, permitting the generation of a peptide [11]. Polyketides (PKs) are a family of compounds made up of units of acyl monomers joined via progressive Claisen condensation catalyzed by the polyketide synthase (PKS) enzyme [11]. In general, NRPs and PKs have a wide range of antagonistic activities. These molecules’ action, along with enzymes which degrade the cell wall, help the plant in its defense against pathogenic attack [12].

*Bacillus* PGPR strains also stimulate plant growth via fixing atmospheric nitrogen (N_2_), solubilizing insoluble phosphates (P) and potassium (K) via secreting organic acids and enzymes and secreting phytohormones, such as indole acetic acid and cytokines, or enzymes which degrade ethylene or γ-aminobutyric acid (GABA) allowing the plant to have more tolerance for biotic and abiotic stress [13,14].

Bacillus-based products are being marketed as plant biostimulants and biopesticides. Examples include RhizoVital^®^ (*Bacillus* *velezensis* FZB42; ABiTEP, GmbH, Berlin, Germany), Serenade^®^ (*B. velezensis* QST713, Bayer, Leverkusen, Germany), Am-ylo-X^®^ WG (*B. amyloliquefaciens* subsp. *plantarum* D747; Certis Europe BV, Utrecht, The Netherlands), RhizoPlus^®^ (*B. subtilis* FZB24; ABiTEP), Sonata^®^ (*B. pumilus* QST2808; AgraQuest, Inc., Davis, CA, USA), Taegro^®^ (*B. subtilis* var. *amyloliquefaciens* FZB24; Novo-zymes Biologicals, Inc., Salem, VA, USA]) [15]. The Bacillus-based biopesticides have been developed mainly for the control of fungal phytopathogens such as *Botrytis,* powdery mildew, *Alternaria*, *Sclerotinia*, *Fusarium* and *Rhizoctonia* [15,16].

In our work, the *B. safensis* RGM 2450 and *B. siamensis* RGM 2529 strains were isolated from the rhizosphere of cardamom crops and Guatemalan native forest, respectively, which presented resilience against biotic and abiotic stress conditions. The species *B. safensis* and *B. siamensis* have previously been described for their biostimulant [17,18,19] and phytopathogen controlling activities [20,21,22,23,24,25]. However, the study of these species’ antagonism has been evaluated in a limited number of phytopathogens, and there are few studies which integrate experimental and genomic analysis of plant-biostimulant and phytopathogen-antagonistic properties to elucidate their PGP action mechanism in economically important crops [26,27]. The hypothesis of the present study was that *B. safensis* RGM 2450 and *B. siamensis* RGM 2529 possess genomic and metabolomic properties involved in antagonistic activity against phytopathogenic fungi and plant-growth promotion. The objectives of this study were to predict the genes involved in the synthesis of PGP factors for *B. safensis* RGM 2450 and *B. siamensis* RGM 2529, evaluate the activity of these strains against four economic impacts relevant to phytopathogenic fungi and evaluate biostimulant activities of both strains in tomato seedlings.

## 2. Materials and Methods

### 2.1. Sample Isolation

The *B. safensis* RGM 2450 strain was isolated from the cardamom cultivation rhizosphere in Cubilhuitz (15°45′59′′, 90°30′04′′), Alta Verapaz, Guatemala. The *B. siamensis* RGM 2529 strain was isolated from the native forest rhizosphere in San Vicente Pacaya (14°24′08′′, 90°36′42′′), Escuintla, Guatemala. Both strains were kept in the Bank of the Chilean Collection of Microbial Genetic Resources at the Agricultural Research Institute (INIA, acronym in Spanish).

The phytopathogenic fungi, Botrytis cinerea, Fusarium oxysporum, Colletotrichum acutatum and Phytophtora cinnamomi, were provided by the Laboratory of Phytopathology at INIA La Platina. They were plated onto potato dextrose agar (PDA) medium in Petri dishes (Ø90 mm) and incubated at 28 °C for for 7–21 days depending on fungal growth speed for use in the antagonist assay.

### 2.2. Genome Sequencing, Assembly and Annotation

Genomic DNA was extracted from the *B. safensis* RGM 2450 and *B. siamensis* RGM 2529 strains using the genomic DNA extraction kit Wizard Genomic DNA (Promega, Madison, WI, USA) following the manufacturer’s instructions. Subsequently, the genomes were sequenced by Novogene Corporation (Davis, CA, USA) using the Illumina sequencing platform (Q30 ≥ 80%) with 150 bp paired-end readings, obtaining 1 Gb of information bases per genome sequenced. The quality of the sequencing obtained was evaluated via FastQC v0.11.7 software [28]. Individual sequences were trimmed off according to their quality using Trimmomatic v0.39 software [29]. The genomes were assembled using SOAPdenovo2 v2.04 software [30]. Assembly quality was evaluated via QUAST v5.0.2 software [31]. Genome annotation was performed via the RAST software based on the RASTtk protocol [32,33]. Functional classification of Clusters of Orthologous Groups (COG) of proteins was based on homology searches using WebMGA [34]. Prediction of the protein signal peptides was carried out with SignalP v5.0 software [35]. Cellular location of proteins was predicted with pSORT software (v.2.0) [36]. The genes encoding PKS and NRPS were predicted with the Antismash v.6.0 bioinformation tool [37,38]. The *B. safensis* RGM 2450 and *B. siamensis* RGM 2529 Whole Genome Shotgun projects have been deposited at DDBJ/ENA/GenBank under the accession numbers JAJQVU000000000 and JAJQVT000000000, respectively. The circular genome visualization of *B. safensis* RGM 2450 and *B. siamensis* RGM 2529 was conducted using DNAPlotter application of the Artemis software ver. 18.0.2 and edited using Inkscape (ver. 1.1.1).

### 2.3. Identification of the B. safensis RGM 2450 and B. siamensis RGM 2529 Strains

Sequences of the gene 16S rRNA from the *B. safensis* RGM 2450 and *B. siamensis* RGM 2529 strains were compared with the curated database of the 16S rRNA gene from GenBank. The sequences obtained from this comparison were aligned using the MUSCLE tool from MEGA v.7.0 software [39]. The evolutionary history was determined using the Neighbor-joining statistical method and the Tamura-3-parameters nucleotide substitution method [40]. The phylogenetic relations hypothesis was supported using 1000 replicates as a bootstrap. To confirm the identification of the *B. safensis* RGM 2450 and *B. siamensis* RGM 2529 strains, we carried out an in silico DNA–DNA hybridization (DDH) analysis using the “Genome-to-genome distance calculator” (GGDC) tool “formula 3” (identities/total length) [41].

### 2.4. Comparative Genomic Analysis

A BLAST score ratio (BSR) test [42] was conducted to compare genes encoding PKS, NRPS, PKS-NRPS, bacteriocins (Table S1) and plant-growth promotion factors (Table S2) between PGPR strain genomes described in this and other studies (Table S3). Each reference gene (Tables S1 and S2) was aligned against each genome with TBLASTN, and the query bit score was tabulated. The BSR value is calculated by dividing the query bit score by the reference bit score, resulting in a BSR value between 0.0 and 1.0 [42]. A score of 1 indicates a perfect match of the reference gene to a query genome, while a score of 0 indicates no BLAST match of the reference gene in the query genome. Values over 0.4 indicate the presence of a gene homologue [42]. The normalized pairs of BSR indices were plotted using R software (version 4.0.).

### 2.5. Evaluation of Antagonistic Activity from Competition

The antagonistic activity of the *B. safensis* RGM 2450 and *B. siamensis* RGM 2529 strains on the phytopathogenic fungi *Botrytis cinerea*, *Fusarium oxysporum*, *Colletotrichum acutatum* and *Phytophtora cinnamomi* was evaluated. The *B. safensis* RGM 2450 and *B. siamensis* RGM 2529 strains were grown in 5 mL of LB medium, respectively, for 16 h at 30 °C and 200 rpm. A total of 100 μL of bacterial culture was sown uniformly on a potato dextrose agar (PDA) plate (BD, Sparks, MD, USA). A 6 mm diameter disc of phytopathogenic fungus was deposited in the center of the inoculated media. The control treatment consisted of a 6 mm disc of phytopathogenic fungi on a PDA plate without bacterial inoculation. Treatments were performed in quintuplicate.

### 2.6. Evaluation of Antagonistic Activity by Diffusible and Volatile Compounds

The antagonistic activity of diffusible compounds secreted by the *B. safensis* RGM 2450 and *B. siamensis* RGM 2529 strains on the aforementioned fungi was evaluated following the methodology described Wang et al. (2018) [43]. The fungi antagonistic impact of volatile organic compounds secreted by the *B. safensis* RGM 2450 and *B. siamensis* RGM 2529 strains was evaluated following the methodology described by Gao et al. (2018) [44]. Treatments were carried out in quintuplicate.

### 2.7. Evaluation of Antagonistic Activity of Phytopathogenic Fungus Growth

The percentage of growth inhibition of phytopathogenic fungus mycelium evaluated was calculated using the PGI formula:

PGI = ((C − T)/C × 100)(1)

where PGI = pathogen mycelial growth inhibition (%); C = diametral pathogen growth (control treatment) and T = diametral pathogen growth (experimental treatment).

### 2.8. Evaluation of Plant-Growth-Promoting Properties

The analysis of the phosphate solubilization of *B. safensis* RGM 2450 and *B. siamensis* RGM 2528 strains was conducted in the Pikovskaya agar medium [45]. The inoculated media were incubated for 7 days at 30 °C. The phosphate solubility potential was calculated on the basis of the phosphate solubilization index [46]. The nitrogen-fixation capacity of these strains was determined using Ashby medium. Bacterial resuspensions of *B. safensis* RGM 2450 and *B. siamensis* RGM 2529 in NaCl 0.9% were adjusted to 1.0 (λ_600_ = 1.0) and inoculated at 2% in liquid Ashby medium. The cultures were incubated at 200 rpm for 72 h at 30 °C. At 0 (recently inoculated), 24, 48 and 72 h, the CFU/mL was evaluated. Concomitantly, a drop of 20 μL of each bacterial resuspension of RGM 2450 and RGM 2529 was sown in Ashby medium agar and incubated at 30 °C for 7 days. Bacterial growth in a liquid and agar medium indicated the capacity to fix N2. RGM 2450 and RGM 2529 strains were inoculated at 2% (turbidity λ_600_ = 1.0–1.2) in LB media supplemented with tryptophan at 1 mg/mL (Sigma Aldrich, Saint Louis, MO, USA). The cultures were incubated at 30 °C for 48 h. Relative IAA concentration in the cultures’ supernatants was determined using the Salkowski method [47]. All tests were performed in triplicate.

### 2.9. Evaluation of Tomato Seedling Growth Promotion

Bacterial suspensions of the *B. safensis* RGM 2450 and *B. siamensis* RGM 2529 strains were grown in LB medium for 20 h at 30 °C. The cultures were washed 3 times and resuspended in a 0.9% NaCl solution supplemented with 0.01% Tween-20 (Sigma Aldrich, USA). The concentration in the resuspension of *B. safensis* RGM 2450 and *B. siamensis* RGM 2529 strains was 10^8^ CFU/mL. Subsequently, 20 seeds were resuspended in each of the following treatments: bacterial resuspension of the *B. safensis* RGM 2450 strain (1), the *B. siamensis* RGM 2529 strain (2), a mixture of the *B. safensis* RGM 2450 and *B. siamensis* RGM 2529 strains (3) and the control treatment (isotonic solution 0.9% NaCl). The seeds from each treatment were sown in seedbeds with a 1:1 (*v*/*v*) mixture of autoclaved peat/vermiculite and incubated at 25 ± 2 °C with a photoperiod of 16/8 h of light/darkness. After 21 days of incubation the following parameters were evaluated: wet weight, dry weight, root length and shoot length in tomato seedlings. To determine dry weight, the seedlings were incubated at 80 °C for 48 h.

### 2.10. Statistical Analysis

According to the experimental design, we carried out the ANOVA LSD Fisher test (α = 0.05) to compare and evaluate antagonistic activities and tomato seedling growth promotion. All results were analyzed and graphed using InfoStat (2020 version) and GraphPad Prim 9 (2020 version), respectively.

## 3. Results

### 3.1. Genome Characteristics and Identification of the B. safensis RGM 2450 and B. siamensis RGM 2529 Strains

The genomes of the *B. safensis* RGM 2450 and *B. siamensis* RGM 2529 strains presented a size of around 3.8–3.9 Mbp which encoded 4139 and 4331 genes, respectively (Table S4). In total, 96% of the genes predicted in the *B. safensis* RGM 2450 and *B. siamensis* RGM 2529 strains encoded proteins, and 41 and 38% of these were classified in COG, respectively (Figure S1). Both strains were distinguishable regarding the % G + C present in their genome, with the *B. safensis* RGM 2450 strain showing 41.3% while *B. siamensis* RGM 2529 showed 45.9% (Table S4).

Phylogenetic analysis of the 16S rRNA gene indicated that the *B. safensis* RGM 2450 and *B. siamensis* RGM 2529 strains belong to the *B. pumilus* and *B. amyloliquefaciens* groups, respectively (Figure S2). We also conducted an in silico DDH. The GGDC showed that the *B. safensis* RGM 2450 strain had the smallest distance from the *Bacillus safensis* FO-36b strain (0.1366), with a DDH estimate of 85.60%. Meanwhile, the RGM 2529 strain had the smallest distance from the *Bacillus siamensis* KCTC 13613 strain (0.1047), with a DDH estimate of 90.70%. A DDH similarity ≥70% indicates that two bacterial strains belong to the same species [48]. These results indicate that the *B. safensis* RGM 2450 and *B. siamensis* RGM 2529 strains belong to the species *B. safensis* and *B. siamensis*, respectively.

### 3.2. Predicting Secondary Metabolites and Extracellular Enzymes

**Figure 1:**
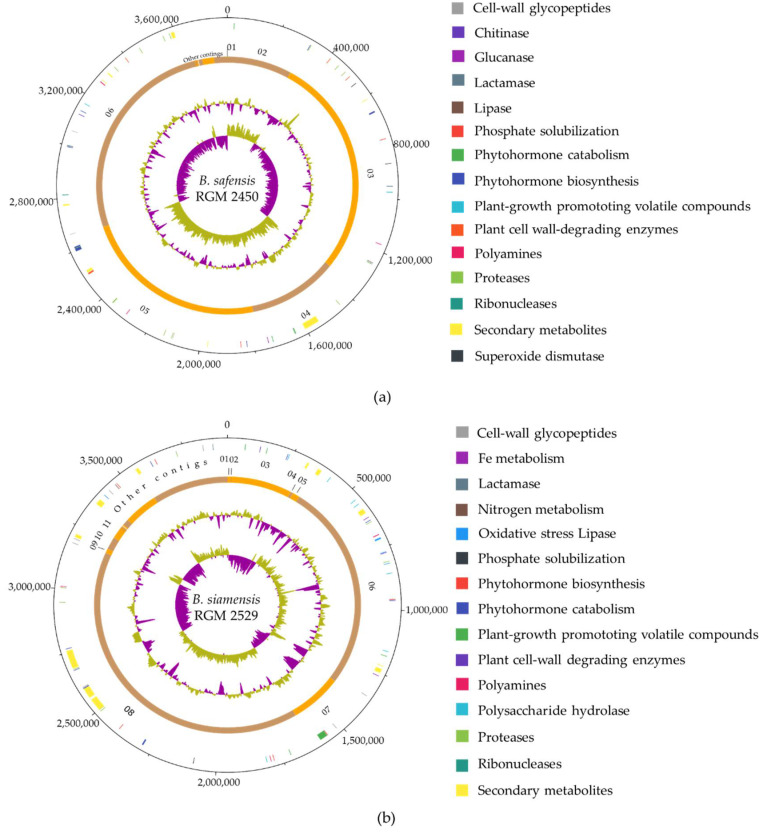
Circular representation of the PGPR bacterial strain genomes. (**a**) *B. safensis* RGM 2450. (**b**) *B. siamensis* RGM 2529. Tracks from outside to inside: circle 1, nucleotide base position (bp) clockwise starting from zero; circle 2, biosynthesis gene clusters or extracellular enzymes genes detected are indicated by colored regions; circle 3, position of DNA contigs, light orange = odd-numbered contigs, dark orange = even-numbered contigs; circle 4, G + C nucleotide content plot, using a 10 kb window size, lime/purple peaks indicate values higher/lower than aver-age G + C content, respectively; circle 5, GC skew plot [(G − C)/(G + C)], using a 10 kb window size, lime/purple peaks indicate values higher/lower than 1, respectively.

**Figure 2:**
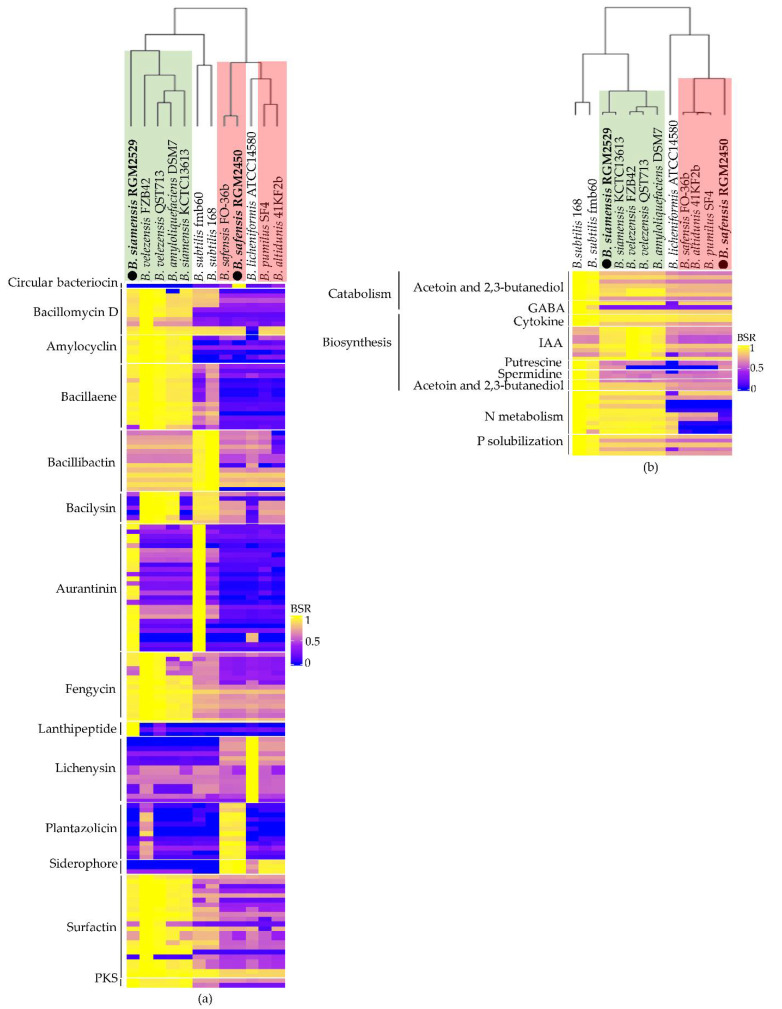
Heat maps displaying the homology levels of genes encoding PKS, NRPS, PKS-NRPS, bacteriocins and plant-growth promotion factors between *B. safensis* RGM 2450 and *B. siamensis* RGM 2529 strains and other *Bacillus* spp. strains described as PGPR. A score of 1 indicates a perfect match, while a score of 0 indicates no BLAST match of a query gene in the reference genome. Values over 0.4 indicate the presence of a homologous gene. (**a**) Comparative analyses of NRPS, PKS and bacteriocin genes participating in the biosynthesis of biocontroller factors. (**b**) Comparative analyses of genes encoding plant-growth promotion factors. The strains that belong to *B. amyloliquefaciens* and *B. pumilus* group are highlighted in green and red, respectively.

Bacteriocins, NRP, PK, NRP-PK hybrids and extracellular enzymes secreted by PGPR strains play a central role in controlling phytopathogenic microorganisms. Based on genome analysis of the *B. safensis* RGM 2450 strain, we predicted the bacteriocins, plantazolicin and bacilysin; two clusters of PKS which participate in the synthesis of alkylpyrone methyl ethers and a type III PKS and two NRPS clusters, one of which is responsible for synthesizing the siderophore bacillibactin and the lipopeptide lichenysin (Figure 1a, Table S5). Most of these gene clusters were also found in *B. safensis* FO-36b, *B. licheniformis* ATCC14580, *B. pumilus* SF4 and *B. altitudines* 41K2b. The genes of a circular bacteriocin and the plantazolicin were exclusively found in the *B. safensis* RGM 2450 strain and phylogenetically closest strain *B. safensis* FO-36b (Figure 2a). The genome of the *B. siamensis* RGM 2529 strain includes the bacteriocin amylocyclin; four clusters of NRPS which synthesize cyclopeptides: fengycin, bacillomicyn D, surfactin and bacillibactin; one cluster of PKS participating in the synthesis of the antibiotic aurantinin and one PKS type III synthase. A gene grouping was also predicted which encodes a hybrid PKS-NRPS that synthesizes the antibiotic bacillaene (Figure 1b, Table S6). Most of these gene clusters were also found in *B. velezensis* FZB42, *B. amyloliquefaciens* DSM7 and *B. siamensis* KCTC 13613 (Figure 2a), while the aurantinin gene cluster was exclusively found in *B. siamensis* RGM 2529, and *B. subtilis* fmb60 strains and the lanthipeptide gene was found only in *B. siamensis* RGM 2529 (Figure 2a).

Furthermore, in the *B. safensis* RGM 2450 strain genome we also predicted 29 enzymes with a signal of localization towards extracellular space (Table S7). The majority of these were proteases (41%), plant cell wall degrading enzymes (21%) and bacteria (10%) (Figure 1a, Table S7). Meanwhile, in the *B. siamensis* RGM 2529 strain genome we predicted 33 extracellular enzymes (Figure 1b), most of which were proteases (24%), polysaccharide hydrolase (21%), plant cell wall degrading enzymes (9%) and bacteria (21%) (Table S8).

### 3.3. Prediction of Pathways Involved in Plant-Growth Promotion

*B. safensis* RGM 2450 and *B. siamensis* RGM 2529 genomes encode the biosynthesis pathway for plant growth factors: IAA, cytokines, polyamines, acetoin and 2,3-butanediol (Figure 1). In both genomes, we found the genes which encode the catabolism pathway for acetoin, 2,3-butanediol and γ-aminobutyric (Tables S3 and S5).

Regarding P solubilization, in the *B. safensis* RGM 2450 and *B. siamensis* RGM 2529 strains possess one and two copies, respectively, of the *phoA* gene which encodes an alkaline phosphatase. This enzyme splits the phosphate groups which are joined to organic compounds, allowing for their bioavailability. Both strains present the *phoR* and *phoP* genes which encode transcriptional regulators of the *phoA* gene. Additionally, the *B. siamensis* RGM 2529 strain genome encodes a phytase. This enzyme sequentially cleaves the six orthophosphate groups joined to the phytate inositol molecule. Both genomes encode the enzymes glucose dehydrogenase and 2-ketogluconate dehydrogenase. These enzymes participate in the production of organic acids including gluconic acid and 2-ketogluconic acid which contribute to inorganic phosphate solubilization (calcium phosphate) (Tables S5 and S6).

Regarding the metabolism of N, in the *B. siamensis* RGM 2529 strain genome we found the cluster gene *ureABC* which encodes a urease. This enzyme converts urea into ammonia (Table S6). We also found the cluster gene *narHIG* that encodes the nitrate reductase, which converts nitrate into nitrite, along with the genes *nasD* and *nasE* which encode the nitrite reductase that takes part in reducing nitrite to ammonia (Table S6).

Comparative genomic analysis indicated that genes involved in plant-growth promotion are widely spread in the different *Bacillus* spp. strains analyzed (Figure 2b). However, genes involved in N metabolism (*ureABC*, *narHIG* and *nasDE*) are present in strains belonging to *B. subtilis* species and *B. amyloliquefaciens* group (Figure 2b).

### 3.4. Evaluation of Strains’ Antagonistic Activity against Phytopathogenic Fungi

**Figure 3:**
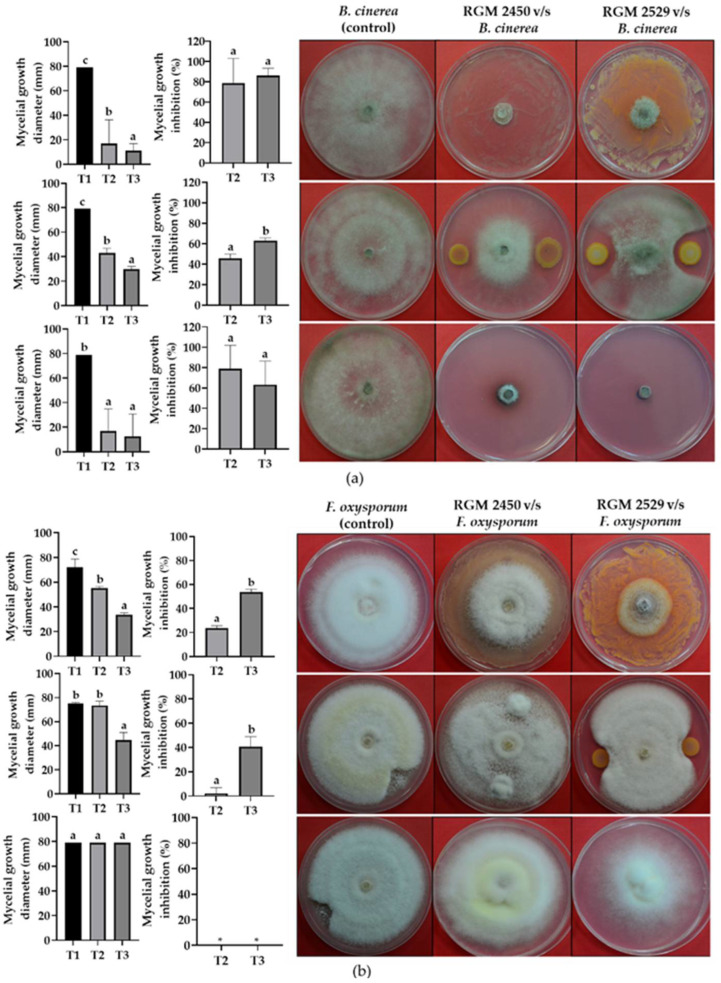

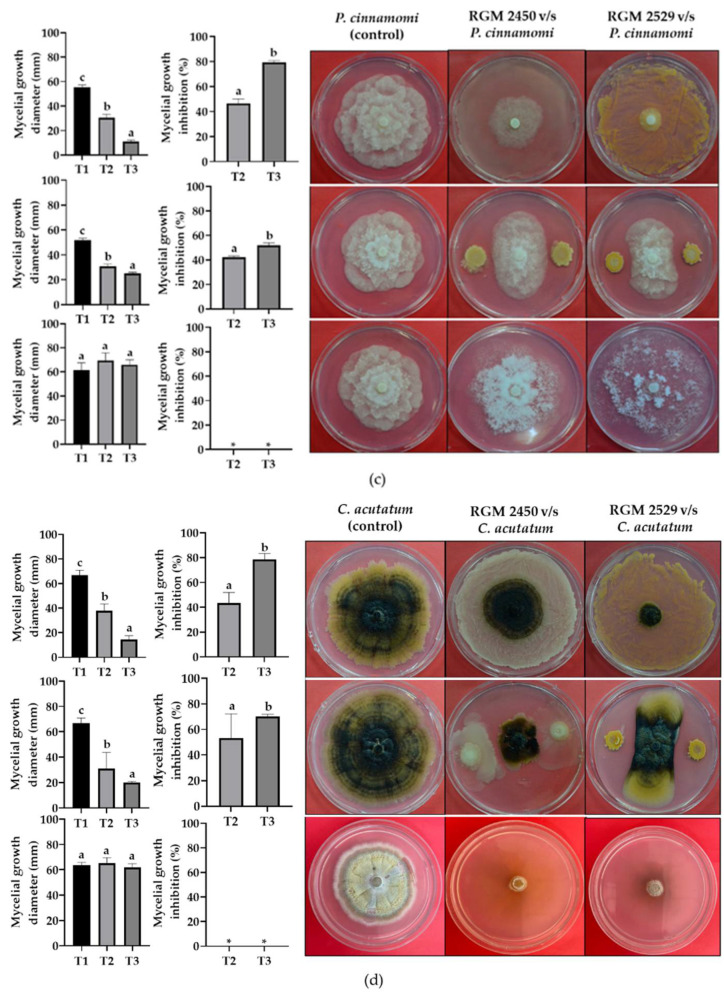
Antagonistic effect of *B. safensis* RGM 2450 and *B. siamensis* RGM 2529 against phytopathogenic fungi (**a**) *B. cinerea*, (**b**) *F. oxysporum*, (**c**) *P. cinnamomi*, (**d**) *C. acutatum*. Treatments: T1, phytopathogenic fungus. T2, strain *B. safensis* RGM 2450 vs. phytopathogenic fungus strain. T3, strain *B. siamensis* RGM 2529 vs. phytopathogenic fungus strain. Letters on the bars represent the comparison using an LSD test (α = 0.05) between treatments. Different letters in each figure represent statistically significant differences. * means that there is no measurable percentage of inhibition.

The % of inhibition of mycelial growth of *B. cinerea*, *F. oxysporum*, *P. cinnamomi* and *C. acutatum* exposed to direct contact with the *B. safensis* RGM 2450 strain compared to the control was 79(±25), 24(±2), 44(±8) and 47(±3)%, respectively (Figure 3). Meanwhile, the mycelial growth inhibition of these fungi exposed to direct contact with the *B. siamensis* RGM 2529 strain was 86(±7), 53(±2), 79(±4) and 80(±1)%, respectively (Figure 3). We also evaluated the capacity of the *B. safensis* RGM 2450 and *B. siamensis* RGM 2529 strains to inhibit fungal growth via secreting diffusible compounds throughout the agar. The results of this test indicated that the *B. safensis* RGM 2450 strain generated a drop in the mycelial growth of *B. cinerea*, *F. oxysporum*, *P. cinnamomi* and *C. acutatum* of 46(±5), 0, 42(±1) and 53(±17)%, respectively, compared to control (Figure 3). The *B. siamensis* RGM 2529 strain generated a decrease of 62(±3), 41(±8), 70(±1) and 52(±2)%, respectively (Figure 3). Concomitantly, we evaluated the capacity to inhibit phytopathogenic fungi via producing volatile compounds. The volatile compounds of the *B. safensis* RGM 2450 and *B. siamensis* RGM 2529 strains generated a drop in *B. cinerea* mycelial growth of 79(±23) and 63(±23)%, respectively, compared to the control (Figure 3a). We observed no decrease in mycelial growth in the other fungi exposed to the volatile compounds secreted by the *B. safensis* RGM 2450 and *B. siamensis* RGM 2529 strains (Figure 3). However, we observed a mycelial growth of *P. cinnamomi* heterogeneously on the agar surface (Figure 3c) compared to the control. In the case of *C. acutatum* we observed mycelial growth primarily under the surface of the agar (Figure 3d) compared with the control treatment.

### 3.5. Evaluation of Plant-Growth-Promoting Properties

We determined the capacity of the *B. safensis* RGM 2450 and *B. siamensis* RGM 2529 strains to fix N_2_, solubilize P and produce IAA. Both strains grew in an Ashby medium (Figure S3). Both strains also presented an increase by one order of magnitude in cellular concentration following 24 h of growth in an Ashby culture. The increase by an order of magnitude in Crops and livestock products. The *B. siamensis* RGM 2529 strain was maintained for up to 72 h of evaluation, while in the case of the *B. safensis* RGM 2450 strain, it decreased by 35% compared to 24 h of growth (Figure S3). On the other hand, the *B. safensis* RGM 2450 and *B. siamensis* RGM 2529 strains presented a phosphorus solubilization index of 1.34 ± 0.17 and 1.73 ± 0.06, respectively (Figure S4). Both strains also showed the capacity to synthesize 8 ± 1 µg/mL of IAA at 48 h of growth in an LB culture medium supplemented with 1 mg/mL of tryptophan.

### 3.6. Evaluation of PGPR Activity in Tomato Seedlings

**Figure 4:**
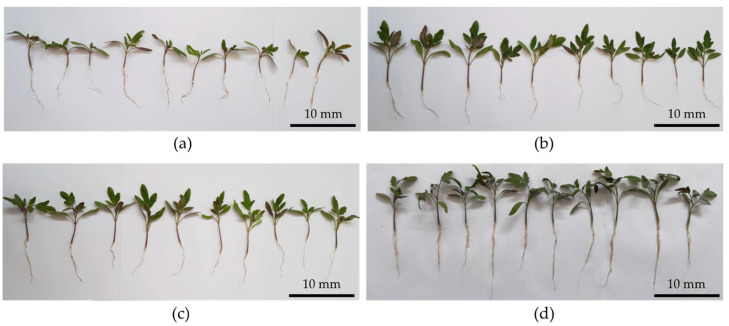
Phenotypes of tomato seedlings from seeds with and without bacterial inoculation. (**a**) Seedling from control treatment (without bacteria). Seedling from treatment inoculated with bacterial resuspension of (**b**) *B. safensis* RGM 2450, (**c**) *B. siamensis* RGM 2529, (**d**) mixture both bacterial strains (1:1). All the treatments were incubated for 21 d at 25 °C, with a 16:8 h light/dark photoperiod.

**Figure 5:**
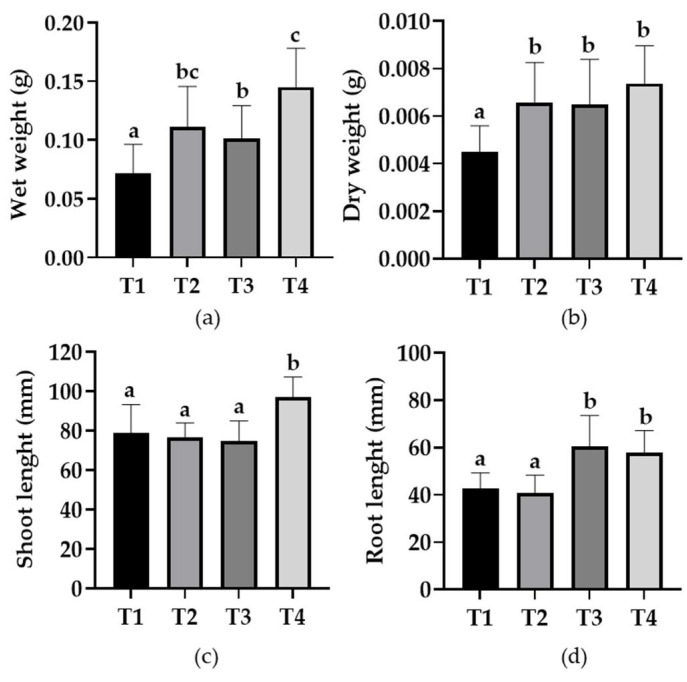
Evaluation of the growth of tomato seedlings from seeds inoculated with bacteria. The evaluation was performed 21 days after seed inoculation. (**a**) Wet weight assessment. (**b**) Dry weight assessment. (**c**) Shoot length assessment. (**d**) Root length assessment. Letters on the bars represent the comparison using an LSD test (α = 0.05) between treatments. Different letters in each figure represent statistically significant differences.

Seedlings of the seeds treated with the bacterial resuspensions showed a significant increase in wet and dry weight compared to the control treatment (Figure 4 and Figure 5). Treatments which were inoculated with the *B. safensis* RGM 2450 strain, the *B. siamensis* RGM 2529 strain and a mixture of both strains showed an increase of between 55, 41 and 103% in wet weight, respectively (Figure 5a), while dry weight evaluations showed an increase of 45, 44 and 63%, respectively, compared to the control (Figure 5b). Regarding shoot length, only the seedlings coming from the seeds treated with the mixture of strains presented an increase of 23%, with the other treatments showing no significant differences compared to the control (Figure 5c). The seedlings from the incubated seeds treated with the *B. siamensis* RGM 2529 strain and mixture of strains showed a root length increase of 42 and 35%, respectively, compared to control (Figure 5d).

## 4. Discussion

The DDH analysis indicated that the *B. safensis* RGM 2450 and *B. siamensis* RGM 2529 strains belong to the *B. safensis* and *B. siamensis* species, respectively. Additionally, the genomes of the strains *B. safensis* RP10 [49], *B. safensis* CFA06 [50] and *B. safensis* ASM189588v1 have a size (3.7 to 3.94 Gpb), GC% (41.4 to 41.7) and number of protein-encoding genes (3781 to 3868) similar to those predicted in the *B. safensis* RGM 2450 strain (Table S4). Similarly, the genomes of the strains of *B. siamensis* KCTC 13613 [51] and SCSIO 05746 [27] have a size (3.8 to 4.27 Mpb), GC% (45.99%) and number of proteins encoding genes (3892 to 4519) similar to those predicted for the *B. siamensis* RGM 2529 strain (Table S4). These results suggest that the genomic information of both strains was obtained in spite of not achieving full genome sequencing or assembly.

The *B. safensis* RGM 2450 strain has the potential to secrete the RP plantazolicin along with the NRPs, bacilysin, bacillibactin and lichenysin (Table S5). Plantazolicin inhibits the growth of Gram-positive bacteria such as *Brevibacillus brevis*, *Micrococcus luteus*, *Paenibacillus granivorans* and *Bacillus anthracis* among other *Bacillus* spp. strains [52,53]. Bacillibactin has a high iron affinity, reducing its bioavailability and thereby limiting surrounding microorganism growth [54,55]. Bacilysin suppresses the biosynthesis of peptidoglycans, which are the main components of bacterial cell walls [56] and also inhibits the production of chitin and mannoproteins in fungal membranes. It has demonstrated antimicrobial activity against *Phytophthora infestans* and *Aspergillus fumigatus*, among other phytopathogens [9,57]. Lichenysin is a cyclical lipoheptapeptide which acts as a powerful surfactant, presenting a critical micellar concentration of 10-22 mg/L [58]. It also acts as a chelant for Ca^2+^ and Mg^2+^ [59]. The predictions of secondary metabolites of the *B. safensis* RGM 2450 strain suggest that its antagonistic activity against phytopathogens is based on competition for macronutrients along with the permeabilization and suppression of membrane biosynthesis.

The genome of the *B. siamensis* RGM 2529 strain encodes an RP and NRP which could participate in the antagonistic activity against phytopathogens (Table S6). Within these peptides, amylocyclin was one of the RPs which was predicted. This circular bacteriocin has been described in the PGPR strain *B. amyloliquefaciens* FZB42 and characterized by its thermostability and its high isoelectric point value [60,61]. It acts against Gram-positive bacteria, causing pores in their membranes. Some of the bacteria which have shown vulnerability to amylocyclin include *Clavibacter michiganensis* NCPPB382 and strains of the species *Bacillus subtillis*, *Micrococcus luteus* and *Paenibacillus granivorans* [60]. *B. siamesis* RGM 2529 also present the *DhbACEBF* operon, which participates in the synthesis of bacillibactin.

We also predicted that the genes participate in surfactin synthesis. This lipopeptide is a biosurfactant that provokes alteration of cell membrane integrity [62]. Surfactin has been described as having antimicrobial activity against *Xanthomonas axonopodis pv. Glycines*, *P. syringae*, *R. solanacearum*, *A. niger*, *B. cinerea*, *F. oxysporum*, *F. solani*, *Monilia fructigena*, *Pennicilium expansum*, *P. italicum* and *R. solani* [63,64,65,66,67,68,69].

The *B. siamensis* RGM 2529 strain genome encodes fengycin and bacillomycin D lipopeptides. The former has been principally described by its strong antifungal activity against various phytopathogenic fungi including *A. solani*, *B. cinerea*, *Fusarium graminearum*, *Fusarium sambucinum*, *F. oxysporum*, *Podosphaera fusca*, *Pythium sulcatum*, *Pythium ultimum*, *R. solani*, *Rhizopus* sp. and *Sclerotinia sclerotiorum*. Bacillomycin D presents activity against *Alternaria alternata*, *A. solani*, *Aspergillus flavus*, *Botryosphaerica ribis*, *C. albicans*, *Cryphonectria parasitica*, *Colletotrichum acutatum*, *Colletotrichum gloesporioides*, *Didymella bryoniae*, *F. graminearum*, *F. oxysporum*, *H. maydis*, *Monilinia fructicola*, *Penicillium expansum*, *Phomopsis gossypii*, *Phytophthora capsici*, *Pyricularia grisea*, *R. solani*, *Sclerotium rolfsii* and *S. sclerotiorum* [9]. Furthermore, Koumoutsi et al. (2004) [70] showed that individual mutants of fengycin and bacillomycin D still largely preserved their capacity to control fungal propagation, but a double mutant which lacked both bacillomycin D and fengycin significantly reduced the inhibition of phytopathogenic fungi, suggesting that both lipopeptides act synergistically.

*B. siamensis* RGM 2529 genome encode PKS which participates in the synthesis of the polyene bacillaene, which presents antimicrobial activity against various bacteria (e.g., *Myxococcus xanthus*, *Staphylococcus aureus*) and fungi (e.g., *Trichoderma* spp., *Fusarium* spp.) (Table S6), along with the gene group that encodes aurantinin. Three types of aurantinin were described in *Bacillus subtillis* fmb60 (A, B and C) which presented antimicrobial activity against *S. aureus* ATCC 25923, *M. luteus* CMCC 28001, *B. pumilus* CMCC 63202, *B. cereus* ATCC 14579, *B. subtilis* ATCC 168, *L. monocytogenes* CICC 21662, *E. faecalis* ATCC 29212 and *P. fluorescens* ATCC 49642 in the same concentration [71]. These compounds produce structural damage in the plasmatic membrane, causing its depolarization and spillage of the intracellular content towards the exterior [71]. The *B. siamensis* RGM 2529 strain could potentially synthesize the three aurantinin types since it presents all the genes which take part in the synthesis of these compounds in the *Bacillus subtillis* fmb60 strain.

Comparative genomic analysis indicates that the profile of antimicrobial compounds synthesized by *B. safensis* RGM 2450 and *B. siamensis* RGM 2529 is similar to strains phylogenetically close to them. However, both strains show a substantially different antimicrobial compound profile. Therefore, this background suggests that the combination of both strains could have a synergistic effect in the control of phytopathogens.

*B. safensis* RGM 2450 and *B. siamensis* RGM 2529 genomes encode extracellular enzymes (Tables S7 and S8) which could participate in the cellular wall degradation of phytopathogenic organisms and plant cell walls. An important number of extracellular proteases were predicted. Proteases secreted by *Bacillus* spp. have been described for their nematicide activity via degrading nematodes’ cuticles. Enzymes which degrade plant cell wall carbohydrates (cellulases, pectinases, xylanases) have been reported in endophytic bacteria [72].

Detecting gene clusters involved in synthesizing antimicrobial factors is consistent with the significant antagonistic activity of *B. safensis* RGM 2450 and *B. siamensis* RGM 2529 against the phytopathogenic fungi *B. cinerea*, *P. cinnamomi* and *C. acutatum* (Figure 3). The *B. siamensis* RGM 2529 strain also was antagonistic to *F. oxysporum* (Figure 3b). These results show important potential for the *B. safensis* RGM 2450 and *B. siamensis* RGM 2529 strains in the pest biocontrol area. *Botrytis cinerea* is the phytopathogenic fungus with the second biggest impact on global agriculture [73]. It is estimated that around 20% of crops harvested worldwide are lost due to *B. cinerea*, generating a loss of between USD 10 and 100 billion annually [74]. The fungus *Colletotrichum* is also one of the most important and widespread global plant pathogens. It particularly attacks crops (anthracnose) in tropical and subtropical regions. The severity of anthracnose disease has led producers to carry out excessive fungicide applications, causing environmental pollution along with increased production costs and in some cases having to write off an entire crop due to this practice failing. *P. cinnamomi* is on the list of the 100 most damaging invasive exotic species in the world from the International Union for Nature Conservation. This fungus causes root rot, which leads to progressive plant decay and death in the case of severe infections. *F. oxysporum* includes a complex of species which jointly infect over 100 different hosts, causing severe losses in crops including melons, tomatoes, cotton and bananas, among others [75]. Subsequent assays in greenhouses and fields will make it possible to evaluate whether the *B. safensis* RGM 2450 and *B. siamensis* RGM 2529 strains can work as biocontrollers for these important agricultural diseases. The comparison of these strains formulated in a mixture or together with other commercial Bacillus-based biopesticides can be used to evaluate the contribution of these strains regarding the current bioproduct market.

The *B. safensis* RGM 2450 and *B. siamensis* RGM 2529 strains also presented genes involved in functions related to plant-growth promotion and colonization. These participate in synthesizing growth-promoting compounds (IAA, cytokines, acetoin, 2,3-butanediol and polyamines) along with genes which participate in GABA degradation which could improve plants’ stress resilience (Tables S5 and S6). We also found genes which participate in phosphorus solubilization and iron acquisition. The genomic comparative analysis suggests these genes are widespread in PGPR belonging to the *Bacillus* genera. Therefore, the experimental performance and comparison of these strains in the same experimental conditions are crucial to evaluate their differential contributions as plant biostimulants.

The genomic prediction findings are consistent with the experimental results. The *B. safensis* RGM 2450 and RGM 2529 strains solubilize P with the latter presenting greater solubilization capacity. Both strains synthesized and secreted IAA in culture media supplemented with the precursor tryptophan. Both strains showed the capacity to grow in a culture medium without an N source, suggesting that they have the capacity to fix N_2_. However, in the genomic analysis we did not detect the gene clusters *nifHDK*, *vnfKGD* and *anfKGD* which encode the three nitrogenase types characterized in the N_2_ fixing microorganisms [76,77]. Therefore, the property of growing without N which *B. safensis* RGM 2450 and *B. siamensis* RGM 2529 present must be mediated by an undescribed mechanism. These experimental studies were complemented with an evaluation of growth promotion in tomato seedlings from seeds inoculated (individually and bacterial mix) (Figure 4 and Figure 5). Treatments inoculated with the strains showed significant increases in wet and dry weight. In the case of the seeds inoculated with the mixtures, there was a rise in wet and dry weight of 103% and 63%, respectively, compared to control, which is a promising result not only because of the weight increase but also because of the hydration percentage which the seedlings presented, which was around 40%. Based on these results, it would be pertinent in future studies to evaluate inoculated plants’ capacity to develop in water stress situations considering the current climate change scenario. The greater root length in tomato seedlings with the *B. siamensis* RGM 2529 strain and mixed strain could be due to the greater availability of macronutrients due to bacterial strain action and the eventual secretion of plant hormones promoting radicular growth (IAA). It should be mentioned that the best phenotype appears in the treatment of the seeds inoculated with the mixed strains compared with the other treatments, suggesting a symbiotic and synergistic relationship between these strains and the plant.

## 5. Conclusions

The genomes of the *B. safensis* RGM 2450 and *B. siamensis* RGM 2529 strains revealed the presence of gene clusters which encode for factors involved in phytopathogen control (NRP, PK and bacteriocins), plant growth stimulation (IAA, cytokines, acetoin, 2,3-butanediol, polyamines, siderophores and P solubilization) and plant tolerance for biotic and abiotic stress (GABA degradation). These findings contribute new knowledge of the genomic properties participating in symbiotic interactions between these strains and the plants.

The genomic comparative analysis herein indicated there is a significative number of homologous genes encoding PKS, NRPS and PKS-NRPS in the strains belonging to same taxonomic group (*B. amyloliquefaciens* or *B. pumilus*), while the almost all genes encoding plant promoting growth factors are widespread in the *Bacillus* strains analyzed in silico. Additionally, this comparative analysis suggests that a mixture of *B.safensis* RGM 2450 and *B. siamensis* RGM 2529 could have a better antagonist performance against phytopathogenic fungi due to producing different microbial compounds.

Experimental tests indicated that these strains’ antagonistic activities against the agriculturally significant phytopathogenic fungi *B. cinerea*, *C. acutatum* and *P. cinnamomi* are due to competition and antibiosis. Future greenhouse and field assays should incorporate at least a commercial *Bacillus*-based biopesticide product to evaluate the contribution of the strains of this study regarding current market options.

Inoculating tomato seeds with a combination of both strains shows a synergistic activity in stimulating growth and tomato seedling hydration. This creates a precedent for larger future studies evaluating this interaction with productivity indicators for this and other crops. These studies should include at least a commercial *Bacillus*-based product to evaluate the real contribution of the mixture formulation of *B. safensis* RGM 2450 and *B. siamensis* RGM 2529 formulation to the currently biostimulant market.

The biostimulant and antagonistic properties against phytopathogens of *Bacillus safensis* RGM 2450 and *Bacillus siamensis* RGM 2529 predicted and evaluated in this study lay the basis for the mechanism by which these strains grant plants resilience to abiotic and biotic stress.

## Data Availability

The *B. safensis* RGM 2450 and RGM 2529 Whole Genome Shotgun projects have been deposited at DDBJ/ENA/GenBank under the accession numbers JAJQVU000000000 and JAJQVT000000000, respectively.

## Supplementary Materials

The following supporting information can be downloaded at: https://www.mdpi.com/article/10.3390/microorganisms10040670/s1, Figure S1: Classification of predicted proteins from *B. safensis* RGM 2450 and *B. siamensis* RGM 2529 according to COG; Figure S2: Phylogenetic relationships of *B. safensis* RGM 2450 and *B. siamensis* RGM 2529 strains based on the *Bacillus* spp. 16S rRNA genes; Figure S3: Evaluation of growth in *B. safensis* RGM 2450 and *B. siamensis* RGM 2529 strains in Ashby medium; Figure S4: Evaluation of the ability of *B. safensis* RGM 2450 and *B. siamensis* RGM 2529 to solubilize inorganic phosphorus; Table S1: Reference genes and proteins involved in the metabolites secondary production; Table S2: Reference genes and proteins involved in plant-growth promotion factors; Table S3: Bacterial strains used in genomic comparative analysis. Table S4: Genome features; Table S5: Comparative analysis of gene clusters involved in plant-growth-promotor factors of *B. safensis* RGM 2450; Table S6: Comparative analysis of gene clusters involved in plant-growth-promotor factors of *B. siamensis* RGM 2529; Table S7: Extracellular enzymes encoded from *B. safensis* RGM 2450 genome; Table S8: Extracellular enzymes encoded from *B. siamensis* RGM 2529 genome.
