# Supplementary material for: Genomic and Experimental Analysis of the Biostimulant and Antagonistic Properties of Phytopathogens of Bacillus safensis and Bacillus siamensis
Source: Microorganisms. 2022 Mar 22;10(4):670. doi: 10.3390/microorganisms10040670 (PMC9024481; doi:10.3390/microorganisms10040670)
Supplement: Supplementary file 1 [file microorganisms-10-00670-s001.zip › microorganisms-1602607 - supplementary/Figure S1.pdf]

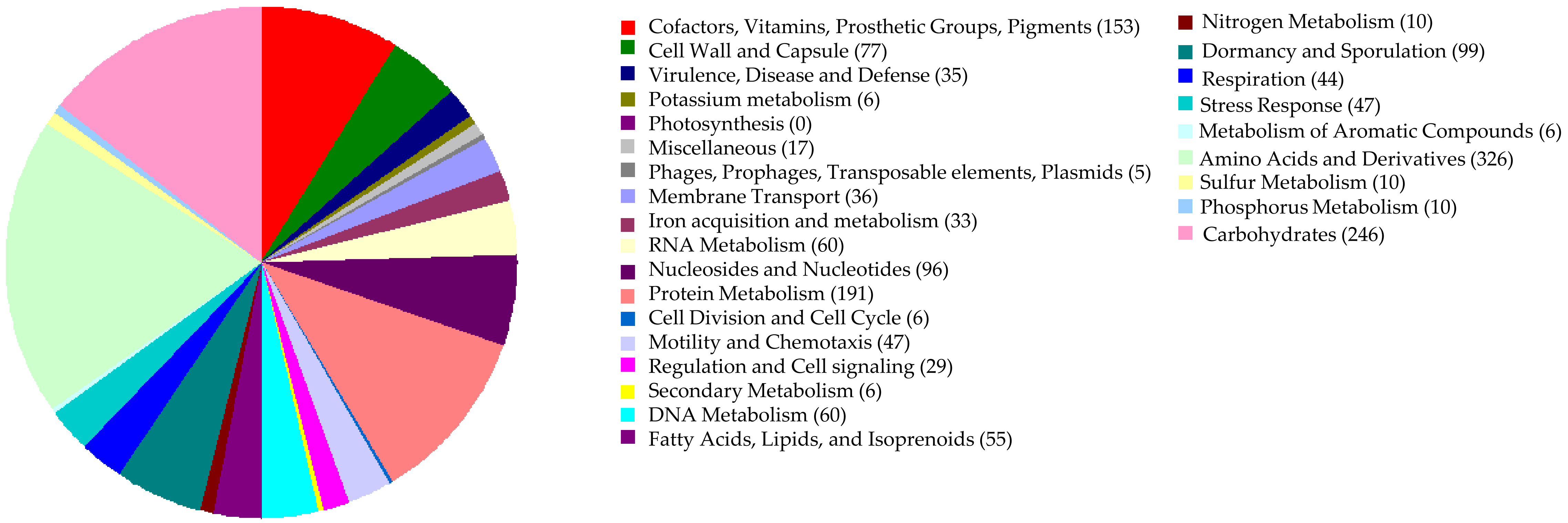

(a)

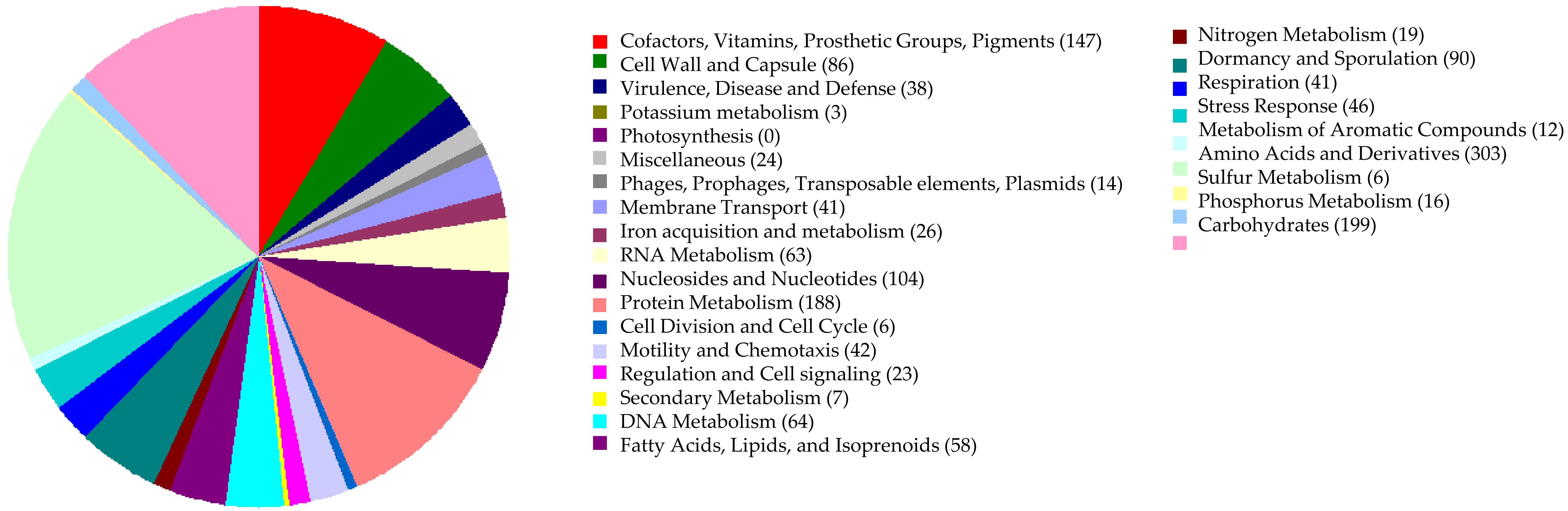

(b)

Figure S1. Classification of predicted proteins from *B. safensis* RGM 2450 and *B. siamensis* RGM 2529 according to COG. (**a**) strain RGM 2450. (**b**) strain RGM 2529.
