# Supplementary material for: Genomic and Experimental Analysis of the Biostimulant and Antagonistic Properties of Phytopathogens of Bacillus safensis and Bacillus siamensis
Source: Microorganisms. 2022 Mar 22;10(4):670. doi: 10.3390/microorganisms10040670 (PMC9024481; doi:10.3390/microorganisms10040670)
Supplement: Supplementary file 1 [file microorganisms-10-00670-s001.zip › microorganisms-1602607 - supplementary/Figure S3 27022022.pdf]

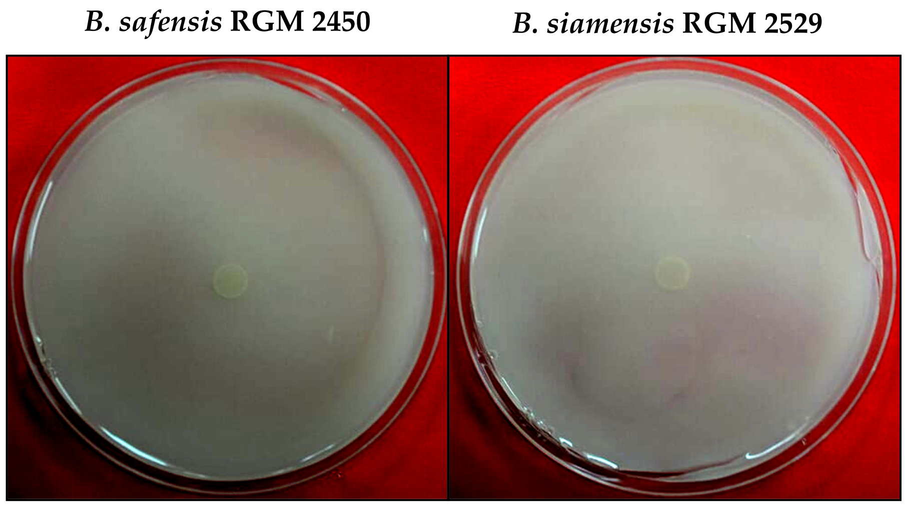

(a)

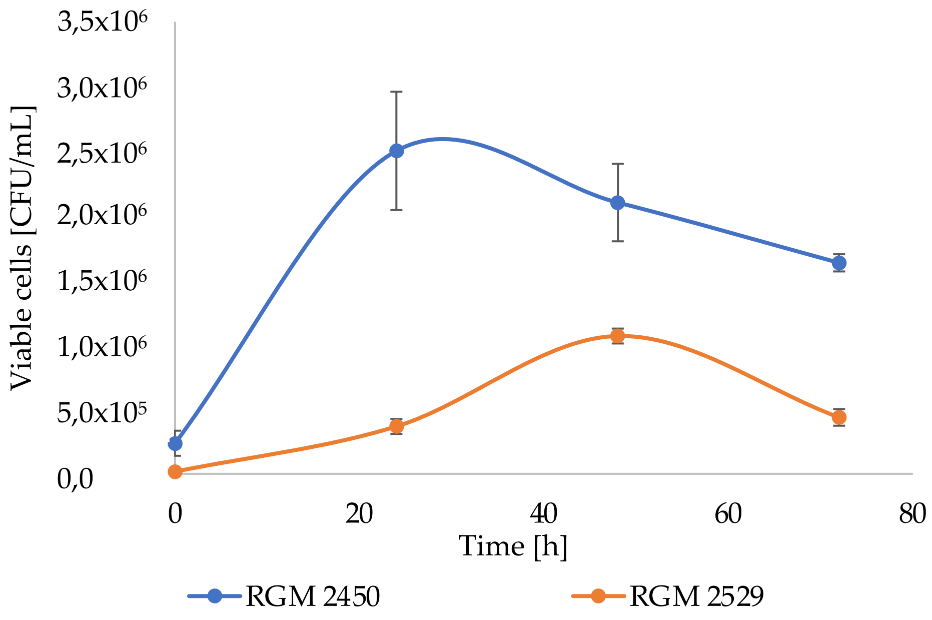

(b)

	Viable cells (CFU/mL)	
Time (h)	RGM 2450	RGM 2529
0	$2,4x10^5 \pm 9,7x10^4$	$1,7x10^4 \pm 4,0x10^3$
24	$2,5x10^6 \pm 4,6x10^5$	$3,7x10^5 \pm 5,8x10^4$
48	$2,1x10^6 \pm 3,0x10^5$	$1,1x10^6 \pm 5,8x10^4$
72	$1,6x10^6 \pm 6,7x10^4$	$4,4x10^5 \pm 6,4x10^4$

(c)

Figure S3. Evaluation of growth in *B. safensis* RGM 2450 and *B. siamensis* RGM 2529 strains in Ashby medium. (**a**) Growth of RGM 2450 and RGM 2529 strains in Ashby agar medium. (**b**) growth curve for RGM 2450 and RGM 2529 strains in Ashby medium. (**c**) Number of viable cells of RGM 2450 and RGM 2529 strains during incubation in Ashby medium.
