# Supplementary material for: Genomic and Experimental Analysis of the Biostimulant and Antagonistic Properties of Phytopathogens of Bacillus safensis and Bacillus siamensis
Source: Microorganisms. 2022 Mar 22;10(4):670. doi: 10.3390/microorganisms10040670 (PMC9024481; doi:10.3390/microorganisms10040670)
Supplement: Supplementary file 1 [file microorganisms-10-00670-s001.zip › microorganisms-1602607 - supplementary/Table S1.pdf]

Table S1. Reference genes and proteins involved in the metabolites secondary production.

Metabolites	Gen Name	Name Protein/Function	Protein Accession N°	Reference
Bacilysin	*ywfG*	YwfG, aminotransferase	RBAM_034880	[1]
	bacE	BacE protein	RBAM_034890	
	bacD	BacD, ligase	RBAM_034900	
	bacC	BacC, alcohol dehydrogenase	RBAM_034910	
	bacB	BacB, isomerase	RBAM_034920	
	bacA	BacA, prephenate decarboxylase	RBAM_034930	
	ywfA	YwfA protein	RBAM_034940	
Bacillibactin	*ald*	L-alanine dehydrogenase	CAB15181.1	[2,3]
	yukJ	Conserved protein of unknown function	CAB15183.2	
	mbtH	Stimulator of DhbF tyrosine adenylation activity	CAX52685.1	
	dhbF	DhbF, Siderophore 2,3-dihydroxybenzoate-glycine-threonine trimeric ester bacillibactin synthetase	CAB15186.3	
	dhbB	DhbB, Isochorismatase (siderophore specific)	CAB15187.1	
	dhbE	DhbE, 2,3-dihydroxybenzoate-AMP ligase	CAB15188.1	
	dhbC	DhbC, Isochorismate synthase (siderophore-specific)	CAB15189.2	
	dhbA	DhbA, 2,3-dihydro-2,3-dihydroxybenzoate dehydrogenase	CAB15190.2	
	besA	Bacillibactin trilactone hydrolase	CAB15191.2	
	yuiH	Putative molybdopterin containing enzyme subunit	CAB15192.1	
	bioYB	Putative biotin transporter	CAB15193.1	
	hisP	Histidine / basic amino acid transporter	CAB15194.1	
	pepA	Cytosol aminopeptidase	CAB15195.1	
Lichenysin	*hyuA1*	Hydantoinase A	AAU39353.1	[4]
	hyuB1	Hydantoinase B	AAU39354.1	
	ND	Putative spermidine/putrescine ABC transporter solute-binding protein	AAU39355.1	
	ND	Putative spermidine/putrescine ABC transporter ATP-binding protein	AAU39356.1	
	ND	Putative spermidine/putrescine ABC transporter permease	AAU39357.1	

	ND	Putative spermidine/putrescine ABC transporter permease	AAU39358.1	
	lchAA	Lichenysin synthase LchAA	AAU39359.1	
	lchAB	Lichenysin synthase LchAB	AAU39360.1	
	lchAC	Lichenysin synthase LchAC	AAU39361.1	
	lchAD	Lichenysin synthase LchAD	AAU39362.1	
	ycxC	Putative transporter YcxC	AAU39363.1	
	ycxD	Putative PLP-dependent transcriptional regulator YcxD	AAU39364.1	
	sfp	4'-phosphopantetheinyl transferase Sfp	AAU39365.1	
	ND	Putative esterase	AU39366.1	
Plantazolicin	*pznF*	Class I SAM-dependent methyltransferase	WP_034619896.1	[5]
	pznK	CPBP family intramembrane metalloprotease	WP_003211749.1	
	pznG	SagB/ThcOx family dehydrogenase	WP_003211436.1	
	pznH	Hypothetical protein	WP_003211025.1	
	pznI	TOMM precursor leader peptide-binding protein	WP_003210769.1	
	pznA	Hypothetical protein	WP_003211085.1	
	pznJ	Plantazolicin family RiPP	WP_003212531.1	
	pznC	Pentapeptide repeat-containing protein	WP_003212115.1	
	pznD	ABC transporter permease	WP_003212220.1	
	pznB	ABC transporter ATP-binding protein	WP_003211121.1	
	pznE	ArsR family transcriptional regulator	WP_034619900.1	
	pznL	Hypothetical protein	WP_003211448.1	
Circular bacteriocin	ND	Uberolysin/carnocyclin family circular bacteriocin	WP_107162921.1	ND
Siderophore	ND	IucA/IucC family siderophore biosynthesis protein	WP_107163851.1	ND
	ND	IucA/IucC family siderophore biosynthesis protein	WP_212040189.1	
	ND	Lysine N(6)-hydroxylase/L-ornithine N(5)-oxygenase family protein	WP_047202330.1	
Surfactin	*yciC*	YciC protein	CAE02619.1	[6]
	yx01	Yx01 protein	CAE02620.1	
	yckC	YckC protein	CAE02621.1	
	yckD	YckD protein	CAE02622.1	

	yczE	YckE protein	CAE02639.1	
	nin	Nin protein	CAE02624.1	
	nucA	NucA	CAE02625.1	
	hxlB	HxlB, hexuloisomerase	CAE02626.1	
	hxlA	HxlA, Hexuloisomerase	CAE02627.1	
	hxlR	HxlR, Transcriptional regulator, HxlR protein	CAE02628.1	
	xy02	Xy02 protein	CAE02629.1	
	srfAA	SrfAA, Surfactin synthetase A	CAE02630.1	
	srfAB	SrfAB, Surfactin synthetase B	CAE02631.1	
	comS	Competence protein S	CAE02632.1	
	srfAC	SrfAC, Surfactin synthetase C	CAE02633.1	
	srfAD	SrfAD, Surfactin synthetase D	CAE02634.1	
	aat	Amino transferase	CAE02635.1	
	ycxc	Transporter	CAE02636.1	
	sfp	Phosphopantetheinyl transferase	CAE02638.1	
	yczE	YczE protein	CAE02639.1	
	yckI	YckI protein	CAE02640.1	
	yckJ	YckJ protein	CAE02641.1	
Fengycin	*dacC*	D-alanyl-D-alanine carboxypeptidase	RBAM_018470	[6]
	fenB	FenB, Fengycin synthetase B	RBAM_018450	
	fenC	FenC, Fengycin sintetase C	RBAM_018440	
	fenA	FenA, Fengycin sintetase A	RBAM_018460	
	fenD	FenD, Fengycin sintetase D	RBAM_018430	
	fenE	FenE, Fengycin sintetase E	RBAM_018420	
	yngL	Proteína YngL	RBAM_018410	
	yngK	Proteína YngK	RBAM_018400	
	yngJ	Acyl-CoA dehydrogenase	RBAM_018390	
	yngI	AMP-binding protein	RBAM_018380	
	yngH	Acetyl-CoA carboxylase biotin carboxylase	RBAM_018370	
	ND	Acetyl-CoA carboxylase biotin carboxyl carrier protein	RBAM_018360	
	yngG	Hydroxymethylglutaryl-CoA lyase	RBAM_018350	

	yngF	Enoyl-CoA hydratase	RBAM_018340	
	yngE	Acyl-CoA carboxylase subunit beta	RBAM_018330	
Bacillaene	*baeS*	Cytochrome P450 'oxidase'	CAG23962.1	[7]
	baeR	Polyketide synthase of type I	CAG23961.2	
	baeN	Hybrid NRPS/PKS protein	CAG23960.2	
	baeM	Polyketide synthase of type I	CAG23959.2	
	baeL	Polyketide synthase of type I	CAG23958.2	
	baeJ	Hybrid NRPS/PKS protein	CAG23957.2	
	baeI	Polyketide biosynthesis enoyl-CoA hydratase	CAG23956.1	
	baeH	Polyketide biosynthesis enoyl-CoA hydratase	CAG23955.1	
	baeG	3-hydroxy-3-methylglutaryl CoA synthase	CAG23954.2	
	acpK	Acyl carrier protein	CAG23953.1	
	baeE	Malonyl-CoA-[acyl-carrier protein] transacylase/oxidoreductase	CAG23952.1	
	baeD	Malonyl-CoA-[acyl-carrier-protein] transacylase	CAG23951.1	
	baeC	Malonyl-CoA-[acyl-carrier-protein] transacylase	CAG23950.2	
	baeB	Hydroxyacylglutathione hydrolase	CAG23949.2	
Aurantinin	ND	Transcription antiterminator	OEI73450.1	[8]
	ND	Hypothetical protein	OEI73451.1	
	ND	Hypothetical protein	OEI73452.1	
	ND	Ubiquinone biosynthesis methyltransferase UbiE	OEI73453.1	
	ND	Hypothetical protein	OEI73454.1	
	ND	S-malonyltransferase	OEI73455.1	
	ND	Poly(3-hydroxyalkanoate) depolymerase	OEI73456.1	
	ND	Polyketide biosynthesis protein	OEI73457.1	
	ND	Alpha/beta hydrolase	OEI73458.1	
	ND	Hypothetical protein	OEI73459.1	
	ND	Hypothetical protein	OEI73460.1	
	ND	Hypothetical protein	OEI73461.1	
	ND	Hypothetical protein	OEI73462.1	
	ND	Hypothetical protein	OEI73463.1	

	ND	Hypothetical protein	OEI73464.1	
	ND	Hypothetical protein	OEI73465.1	
	ND	Hypothetical protein	OEI73466.1	
	ND	Polyketide beta-ketoacyl:ACP synthase	OEI73467.1	
	ND	Enoyl-CoA hydratase	OEI73469.1	
	ND	Enoyl-CoA hydratase	OEI73470.1	
	ND	Hypothetical protein	OEI73471.1	
	ND	Hypothetical protein	OEI73472.1	
	ND	Glycosyl transferase	OEI73473.1	
	ND	Hypothetical protein	OEI73474.1	
	ND	Hypothetical protein	OEI73475.1	
	ND	MATE family efflux transporter	OEI73476.1	
	ND	Methyltransferase type 11	OEI73477.1	
Bacillomycin D	*yxjC*	GntP family permease	RBAM_018230	[6]
	scoA	CoA transferase subunit A	RBAM_018220	
	scoB	CoA transferase subunit B	RBAM_018210	
	yxjF	3-hydroxybutyrate dehydrogenase	RBAM_018200	
	bmyD	ACP S-malonyltransferase	RBAM_018190	
	bmyA	Bacillomycin D synthetase A	RBAM_018180	
	bmyB	Bacillomycin D synthetase B	RBAM_018170	
	bmyC	Bacillomycin D synthetase C	RBAM_018160	
	xynD	Carbohydrate-binding protein	RBAM_018150	
	ynfF	Glucuronoxylanase	RBAM_018140	
Amylocyclin	*acnB*	Transmembrane protein, essential for synthesis	RBAM_029190	[9]
	acnA	Precursor of circular bacteriocin	RBAM_029200	
	acnC	Processing, maturation, and self-immunity	RBAM_029210	
	acnD	ABC transporter	RBAM_029220	
	acnE	Integral membrane protein,	RBAM_029230	
	acnF	Transmembrane protein	RBAM_029240	
Polyketide synthase	ND	Type III polyketide synthase/Predicted naringenin-chalcone synthase	WP_039179425.1	ND

Polyketide synthase	ND	polyketide beta-ketoacyl:ACP synthase	WP_047476665.1	ND
Lanthipeptide	ND	lanthionine synthetase C	WP_041850824.1	ND
	ND	ABC transporter ATP-binding protein/permease	WP_047474965.1	
	ND	lantibiotic dehydratase	WP_047474967.1	

ND, no described.

References

1. Wu, L.; Wu, H.; Chen, L.; Xie, S.; Zang, H.; Borriss, R.; Gao, X. Bacilysin from Bacillus amyloliquefaciens FZB42 Has Specific Bactericidal Activity against Harmful Algal Bloom Species. *Appl. Environ. Microbiol.* **2014**, *80*, 7512–7520. https://doi.org/10.1128/aem.02605-14.
2. Kunst, F.; Ogasawara, N.; Moszer, I.; Albertini, A.M.; Alloni, G.; Azevedo, V.; Bertero, M.G.; Bessieres, P.; Bolotin, A.; Borchert, S.; et al. The complete genome sequence of the Gram-positive bacterium Bacillus subtilis. *Nature* **1997**, *390*, 249–256. https://doi.org/10.1038/36786.
3. Barbe, V.; Cruveiller, S.; Kunst, F.; Lenoble, P.; Meurice, G.; Sekowska, A.; Vallenet, D.; Wang, T.; Moszer, I.; Médigue, C.; et al. From a consortium sequence to a unified sequence: The Bacillus subtilis 168 reference genome a decade later. *Microbiology* **2009**, *155*, 1758–1775. https://doi.org/10.1099/mic.0.027839-0.
4. Veith, B.; Herzberg, C.; Steckel, S.; Feesche, J.; Maurer, K.H.; Ehrenreich, P.; Bäumer, S.; Henne, A.; Liesegang, H.; Merkl, R.; et al. The Complete Genome Sequence of Bacillus licheniformis DSM13, an Organism with Great Industrial Potential. *J. Mol. Microbiol. Biotechnol.* **2004**, *7*, 204–211. https://doi.org/10.1159/000079829.
5. Molohon, K.J.; Melby, J.O.; Lee, J.; Evans, B.S.; Dunbar, K.L.; Bumpus, S.B.; Kelleher, N.L.; Mitchell, D.A. Structure Determination and Interception of Biosynthetic Intermediates for the Plantazolicin Class of Highly Discriminating Antibiotics. *ACS Chem. Biol.* **2011**, *6*, 1307–1313. https://doi.org/10.1021/cb200339d.
6. Koumoutsi, A.; Chen, X.-H.; Henne, A.; Liesegang, H.; Hitzeroth, G.; Franke, P.; Vater, J.; Borriss, R. Structural and Functional Characterization of Gene Clusters Directing Nonribosomal Synthesis of Bioactive Cyclic Lipopeptides in Bacillus amyloliquefaciens Strain FZB42. *J. Bacteriol.* **2004**, *186*, 1084–1096. https://doi.org/10.1128/jb.186.4.1084-1096.2004.
7. Moldenhauer, J.; Götz, D.C.G.; Albert, C.R.; Bischof, S.K.; Schneider, K.; Süssmuth, R.D.; Engeser, M.; Gross, H.; Bringmann, G.; Piel, J. The Final Steps of Bacillaene Biosynthesis in Bacillus amyloliquefaciens FZB42: Direct Evidence for β,γ Dehydration by a trans-Acyltransferase Polyketide Synthase. *Angew. Chem. Int. Ed.* **2010**, *49*, 1465–1467. https://doi.org/10.1002/anie.200905468.
8. Idris, E.E.; Iglesias, D.J.; Talon, M.; Borriss, R. Tryptophan-Dependent Production of Indole-3-Acetic Acid (IAA) Affects Level of Plant Growth Promotion by Bacillus amyloliquefaciens FZB42. *Mol. Plant-Microbe Interact.* **2007**, *20*, 619–626. https://doi.org/10.1094/mpmi-20-6-0619.
9. Scholz, R.; Vater, J.; Budiharjo, A.; Wang, Z.; He, Y.; Dietel, K.; Schwecke, T.; Herfort, S.; Lasch, P.; Borriss, R. Amylocyclicin, a Novel Circular Bacteriocin Produced by Bacillus amyloliquefaciens FZB42. *J. Bacteriol.* **2014**, *196*, 1842–1852. https://doi.org/10.1128/jb.01474-14.
