# Supplementary material for: Genomic and Experimental Analysis of the Biostimulant and Antagonistic Properties of Phytopathogens of Bacillus safensis and Bacillus siamensis
Source: Microorganisms. 2022 Mar 22;10(4):670. doi: 10.3390/microorganisms10040670 (PMC9024481; doi:10.3390/microorganisms10040670)
Supplement: Supplementary file 1 [file microorganisms-10-00670-s001.zip › microorganisms-1602607 - supplementary/Table S2.pdf]

Table S2. Reference genes and proteins involved in plant growth promotion factors.

Type of Factor	Factor	Gen Name	Function	Protein Accession N°	Reference
Phytohormons biosynthesis	Indol-3-acetic acid	*trpE*	anthranilate synthase	CAL26225.1	[1]
		trpD	anthranilate phosphoribosyltransferase	CAL26226.1	
		trpC	indole-3-glycerol-phosphate synthase	WP_012117859.1	
		trpF	N-(5'-phosphoribosyl)anthranilate isomerase	WP_012117858.1	
		trpB	tryptophan synthase subunit beta	WP_012117857.1	
		trpA	tryptophan synthase subunit alpha	WP_012117856.1	
		ysnE	Putative IAA-acetyl-transferase	WP_012118742.1	
		yhcX	Nitrilase	CAL26199.1	
	Cytokinin	*miaA*	tRNA dimethylallyltransferase	NP_389615	[2]
		miaB	tRNA-2-methylthio-N(6)-dimethylallyladenosine synthase	NP_389583	
		yvdD	cytokinin riboside 5′-monofosfato fosforibohidrolase	NP_391344.1	
Polyamines biosynthesis	Putrescine	*speA*	Arginine decarboxylase	NP_389346.1	
		speB	Agmatinase	NP_391629.1	
	Spermidine	*speD*	S-adenosylmethionine decarboxylase	NP_390779.2	
		speE	Polyamine aminopropyltransferase	NP_391630.1	
Plant growth promotor volatil compound	Acetoin and 2,3-butanediol	*alsS*	Acetolactate synthase	ILVX_BACSU	[3]
		alsD	Alpha-acetolactate decarboxylase	ALDC_BACSU	
		bdhA	(R,R)-butanediol dehydrogenase	BDHA_BACSU	
		alsR	HTH-type transcriptional regulator AlsR	ALSR_BACSU	
Phytohormon catabolism	Acetoin and 2,3-butanediol	*acuA*	Acetoin utilization protein AcuA	ACUA_BACSU	
		acuB	Acetoin utilization protein AcuB	ACUB_BACSU	
		acuC	Acetoin utilization protein AcuC	ACUC_BACSU	
		acoA	2,6-dichlorophenolindophenol oxidoreductase subunit alpha	ACOA_BACSU	
		acoB	2,6-dichlorophenolindophenol oxidoreductase subunit beta	ACOB_BACSU	
		acoC	Acetoin dehydrogenase E2 component	ACOC_BACSU	

	bdhA	(R,R)-butanediol dehydrogenase	BDHA_BACSU	
GABA	*gadP*	GABA permease	BSU06310	[4]
	gadD	Succinate-semialdehyde dehydrogenase	BSU03910	
	gadT	4-aminobutyrate aminotransferase	BSU03900	
Phosphate solubilization	phoR	Alkaline phosphatase synthesis	PHOR_BACSU	[5]
	phoP	Alkaline phosphatase synthesis transcriptional regulatory protein	PHOP_BACSU	
	phoA	Alkaline phosphatase	PPB4_BACSU	
	gdh	Glucose 1-dehydrogenase	DHG_BACSU	
	yvcT	2-ketogluconate reductase	TKRA_BACSU	
Nitrogen metabolims	*ureA*	Urea amidohydrolase subunit gamma	URE3_BACSU	[6]
	ureB	Urease subunit beta	URE2_BACSU	
	ureC	Urease subunit alpha	URE1_BACSU	
	nasD	Nitrite reductase	NASD_BACSU	
	nasE	Assimilatory nitrite reductase [NAD(P)H] small subunit	NASE_BACSU	
	narG	Nitrate reductase alpha chain	NARG_BACSU	
	narI	Nitrate reductase gamma chain	NARI_BACSU	
	narH	Nitrate reductase beta chain	NARH_BACSU	

References

1. Idris, E.E.; Iglesias, D.J.; Talon, M.; Borriss, R. Tryptophan-Dependent Production of Indole-3-Acetic Acid (IAA) Affects Level of Plant Growth Promotion by Bacillus amyloliquefaciens FZB42. *Mol. Plant-Microbe Interact.* **2007**, *20*, 619–626. https://doi.org/10.1094/mpmi-20-6-0619.
2. Borriss, R.; Danchin, A.; Harwood, C.R.; Médigue, C.; Rocha, E.P.; Sekowska, A.; Vallenet, D. Bacillus subtilis,the model Gram-positive bacterium: 20 years of annotation refinement. *Microb. Biotechnol.* **2017**, *11*, 3–17. https://doi.org/10.1111/1751-7915.13043.
3. Ryu, C.-M.; Farag, M.A.; Hu, C.-H.; Reddy, M.S.; Wei, H.-X.; Paré, P.W.; Kloepper, J.W. Bacterial volatiles promote growth in Arabidopsis. *Proc. Natl. Acad. Sci. USA* **2003**, *100*, 4927–4932. https://doi.org/10.1073/pnas.0730845100.
4. Belitsky, B.R.; Sonenshein, A.L. GabR, a member of a novel protein family, regulates the utilization of gamma-aminobutyrate in Bacillus subtilis. *Mol. Microbiol.* **2002**, *45*, 569–583. https://doi.org/10.1046/j.1365-2958.2002.03036.x.
5. Hulett, F.M.; Kim, E.E.; Bookstein, C.; Kapp, N.V.; Edwards, C.W.; Wyckoff, H.W. Bacillus subtilis alkaline phosphatases III and IV. Cloning, sequencing, and comparisons of deduced amino acid sequence with Escherichia coli alkaline phosphatase three-dimensional structure. *J. Biol. Chem.* **1991**, *266*, 1077–1084.
6. Kim, J.K.; Mulrooney, S.B.; Hausinger, R.P. Biosynthesis of Active Bacillus subtilis Urease in the Absence of Known Urease Accessory Proteins. *J. Bacteriol.* **2005**, *187*, 7150–7154. https://doi.org/10.1128/jb.187.20.7150-7154.2005.
