# Supplementary material for: Genomic and Experimental Analysis of the Biostimulant and Antagonistic Properties of Phytopathogens of Bacillus safensis and Bacillus siamensis
Source: Microorganisms. 2022 Mar 22;10(4):670. doi: 10.3390/microorganisms10040670 (PMC9024481; doi:10.3390/microorganisms10040670)
Supplement: Supplementary file 1 [file microorganisms-10-00670-s001.zip › microorganisms-1602607 - supplementary/Table S3.pdf]

Table S3. Bacterial strains used in genomic comparative analysis.

Strains	Accession Number Genebank	Ref.
Bacillus safensis RGM 2450	JAJQVU000000000	This study
Bacillus siamenesis RGM 2529	JAJQVT000000000	This study
Bacillus altitudinis 41KF2b	NZ_ASJC00000000.1	[1]
Bacillus amyloliquefaciens DSM7	NC_014551.1	[2]
Bacillus licheniformis ATCC 14580	CP000002.3	[3]
Bacillus pumilus SF4	CP047089.1	[4]
Bacillus safensis FO-36b	CP010405.1	[5]
Bacillus siamensis KCTC 13613	NZ_AJVF00000000.1	[6]
Bacillus subtilis 168	NC_000964.3	[7]
Bacillus subtilis fmb60	LYMC00000000.1	[8]
Bacillus velezensis FZB42	NC_009725.2	[9]
Bacillus velezensis QST713	NZ_CP025079.1	[10]

References

1. Shivaji, S.; Chaturvedi, P.; Suresh, K.; Reddy, G.S.N.; Dutt, C.B.S.; Wainwright, M.; Narlikar, J.V.; Bhargava, P.M. Bacillus aerius sp. nov., Bacillus aerophilus sp. nov., Bacillus stratosphericus sp. nov. and Bacillus altitudinis sp. nov., isolated from cryogenic tubes used for collecting air samples from high altitudes. *Int. J. Syst. Evol. Microbiol.* **2006**, *56*, 1465–1473. https://doi.org/10.1099/ijs.0.64029-0.
2. Rückert, C.; Blom, J.; Chen, X.; Reva, O.; Borriss, R. Genome sequence of B. amyloliquefaciens type strain DSM7T reveals differences to plant-associated B. amyloliquefaciens FZB42. *J. Biotechnol.* **2011**, *155*, 78–85. https://doi.org/10.1016/j.jbiotec.2011.01.006.
3. Rey, M.W.; Ramaiya, P.; A Nelson, B.; Brody-Karpin, S.D.; Zaretsky, E.J.; Tang, M.; De Leon, A.L.; Xiang, H.; Gusti, V.; Clausen, I.G.; et al. Complete genome sequence of the industrial bacterium Bacillus licheniformis and comparisons with closely related Bacillus species. *Genome Biol.* **2004**, *5*, r77–r77. https://doi.org/10.1186/gb-2004-5-10-r77.
4. Iqbal, S.; Vollmers, J.; Janjua, H. Genome Mining and Comparative Genome Analysis Revealed Niche-Specific Genome Expansion in Antibacterial *Bacillus pumilus* Strain SF-4. *Genes* **2021**, *12*, 1060. https://doi.org/10.3390/genes12071060.
5. Tirumalai, M.R.; Stepanov, V.G.; Wünsche, A.; Montazari, S.; Gonzalez, R.O.; Venkateswaran, K.; Fox, G.E. Bacillus safensis FO-36b and Bacillus pumilus SAFR-032: A whole genome comparison of two spacecraft assembly facility isolates. *BMC Microbiol.* **2018**, *18*, 1–16. https://doi.org/10.1186/s12866-018-1191-y.
6. Jeong, H.; Jeong, D.-E.; Kim, S.H.; Song, G.C.; Park, S.-Y.; Ryu, C.-M.; Park, S.-H.; Choi, S.-K. Draft Genome Sequence of the Plant Growth-Promoting Bacterium Bacillus siamensis KCTC 13613 T. *J. Bacteriol.* **2012**, *194*, 4148–4149. https://doi.org/10.1128/jb.00805-12.
7. Borriss, R.; Danchin, A.; Harwood, C.R.; Médigue, C.; Rocha, E.P.; Sekowska, A.; Vallenet, D. Bacillus subtilis,the model Gram-positive bacterium: 20 years of annotation refinement. *Microb. Biotechnol.* **2017**, *11*, 3–17. https://doi.org/10.1111/1751-7915.13043.
8. Yang, J.; Zhu, X.; Cao, M.; Wang, C.; Zhang, C.; Lu, Z.; Lu, F. Genomics-Inspired Discovery of Three Antibacterial Active Metabolites, Aurantinins B, C, and D from Compost-Associated Bacillus subtilis fmb60. *J. Agric. Food Chem.* **2016**, *64*, 8811–8820. https://doi.org/10.1021/acs.jafc.6b04455.
9. Fan, B.; Wang, C.; Song, X.; Ding, X.; Wu, L.; Wu, H.; Gao, X.; Borriss, R. Bacillus velezensis FZB42 in 2018: The Gram-Positive Model Strain for Plant Growth Promotion and Biocontrol. *Front. Microbiol.* **2018**, *9*, 2491. https://doi.org/10.3389/fmicb.2018.02491.
10. Pandin, C.; Le Coq, D.; Deschamps, J.; Védie, R.; Rousseau, T.; Aymerich, S.; Briandet, R. Complete genome sequence of Bacillus velezensis QST713: A biocontrol agent that protects Agaricus bisporus crops against the green mould disease. *J. Biotechnol.* **2018**, *20*, 10–19. https://doi.org/10.1016/j.jbiotec.2018.04.014.
