# Supplementary material for: Genomic and Experimental Analysis of the Biostimulant and Antagonistic Properties of Phytopathogens of Bacillus safensis and Bacillus siamensis
Source: Microorganisms. 2022 Mar 22;10(4):670. doi: 10.3390/microorganisms10040670 (PMC9024481; doi:10.3390/microorganisms10040670)
Supplement: Supplementary file 1 [file microorganisms-10-00670-s001.zip › microorganisms-1602607 - supplementary/Table S4.pdf]

Table S4. Genome features.

	B. safensis RGM 2450		*B. siamensis* RGM 2529	
**Attribute**	**Value**	**%**	**Value**	**%**
Genome size (bp)	3,798,090	100	3,916,442	100
DNA coding (bp)	3,372,920	89	3,492,149	89
DNA G + C (bp)	1,568,611	41.3	1,797,647	45.9
Scaffold de DNA	6	-	11	-
N° total de genes	4139	100.0	4.331	100
L50	4	-	9	-
Protein coding genes	3982	96	4.174	96
RNA genes	79	1.9	87	2
Genes assigned to COGs	1710	41	1666	38
Protein with signal peptides	373	9.0	367	8.4
Protein with extracellular domain	81	1.9	98	2.3
