# Supplementary material for: Genomic and Experimental Analysis of the Biostimulant and Antagonistic Properties of Phytopathogens of Bacillus safensis and Bacillus siamensis
Source: Microorganisms. 2022 Mar 22;10(4):670. doi: 10.3390/microorganisms10040670 (PMC9024481; doi:10.3390/microorganisms10040670)
Supplement: Supplementary file 1 [file microorganisms-10-00670-s001.zip › microorganisms-1602607 - supplementary/Table S5.pdf]

Table S5. Comparative analysis of gene clusters involved in plant growth promotor factors of *B. safensis* RGM 2450.

Type Factor	Factor	Location	Cluster Position	Identity	
				**PGPR Strain**	**%**
Secondary metabolites	Bacilysin	Scaffold 6	657131-660805	*B. velezensis* QST 713	64
	Bacillibactin	Scaffold 6	936058-947791	*B. velezensis* QST 714	63
			127070-121285		
	lichenysin	Scaffold 4	188228-245982	*B. licheniformis* DSM 13	60
	Plantazolicin	Scaffold 5	728351-736760	*B. velezensis* QST 713	63
Phytohormon biosynthesis	Indol-3-acetic acid	Scaffold 3	361955-358375	*B. velezensis* FZB421	69
		Scaffold 3	487657-488112		
		Scaffold 3	47276-48814		
	Cytokinin	Scaffold 5	834710-818990	*Priestia megaterium* STB1	67
		Scaffold 6	344079-344657		
		Scaffold 3	14793-17936		
Polyamines	Putrescine	Scaffold 5	517914-519404	*Priestia megaterium* STB1	80
		Scaffold 6	616820-617692		
	Spermidine	Scaffold 3	871466-871846	*Priestia megaterium* STB1	85
		Scaffold 6	617762-618592		
Plant growth promotor volatil compound	Acetoin and 2,3-butanediol	Scaffold 6	517450-520994	*B. subtilis* 168	71
		Scaffold 3	671282-672322		
Phytohormon catabolism	GABA	Scaffold 2	24124-25512	*B. subtilis* 168	78
		Scaffold 4	283557-284879		
		Scaffold 4	286264-287658		
	Acetoin and 2,3-butanediol	Scaffold 4	371583-374775	*B. subtilis* 168	81
		Scaffold 3	671282-672322		
		Scaffold 3	951745-954215		
Phosphate solubilization	Alkaline phophatase	Scaffold 3	877458-880200	*B. subtilis* 168	66
		Scaffold 3	880193-880921		
		Scaffold 5	69850-71214		
	Organic acid	Scaffold 3	468975-469760	*B. subtilis* 168	66
		Scaffold 6	347954-348931		
