# Supplementary material for: Genomic and Experimental Analysis of the Biostimulant and Antagonistic Properties of Phytopathogens of Bacillus safensis and Bacillus siamensis
Source: Microorganisms. 2022 Mar 22;10(4):670. doi: 10.3390/microorganisms10040670 (PMC9024481; doi:10.3390/microorganisms10040670)
Supplement: Supplementary file 1 [file microorganisms-10-00670-s001.zip › microorganisms-1602607 - supplementary/Table S6.pdf]

Table S6. Comparative analysis of gene clusters involved in plant growth promotor factors of *B. siamensis* RGM 2529.

Type Factor	Factor	Location	Cluster Position	Identity	
				**PGPR Strain**	**%**
Secondary metabolites	Bacillibactin	Scaffold 6	895012-878233	*B. velezensis* QST 713	92
	Surfactin	Scaffold 6	2-15407	*B. velezensis* QST 713	93
			227358-247054		
	Fengycin	Scaffold 8	874448-923913	*B. velezensis* QST 713	92
	Bacillaene	Scaffold 8	1061005-1133351	*B. velezensis* QST 713	92
	Aurantinin	Contig 419	2-22820	*B. subtilis* fmb60	97
		Contig 471	1380-13853		
		Scaffold 4	1-7960		92
		Scaffold 9	9725-23086		86
	Bacillomycin D	Scaffold 8	932676-977313	*B. velezensis* QST 714	86
	Amylocyclin	Scaffold 6	866517-870687	*B. velezensis* QST 715	77
Phytohormon byosisnthesis	Indol-3-acetic acid	Scaffold 8	667442-661531	*B. velezensis* FZB421	93
		Scaffold 6	279225-279683		
		Contig 471	64800-66338		
	Cytokinin	Scaffold 8	1052977-1053921	*Priestia megaterium* STB1	67
		Scaffold 8	1140820-1142349		
		Scaffold 6	606838-607413		
		Scaffold 3	132047-135232		
Polyamines	Putrescine	Scaffold 8	1385796-1387268	*Priestia megaterium* STB1	79
		Scaffold 6	343797-344669		
	Spermidin	Scaffold 8	169859-170239	*Priestia megaterium* STB1	84
		Scaffold 6	342908-343738		
Plant growth promotor volatil compound	Acetoin and 2,3-butanediol	Scaffold 6	460390-463993	*B. subtilis* 168	80
		Scaffold 3	217726-218766		
Phytohormon catabolism	GABA	Scaffold 7	187922-218766	*B. subtilis* 168	87
		Scaffold 3	212141-213526		
	Acetoin and 2,3-butanediol	Scaffold 8	92563-94395	*B. subtilis* 168	81
		Scaffold 3	49196-52437		
Phosphate solubilization	Alkaline phophatase	Scaffold 8	158106-160557	*B. subtilis* 168	79
		Conting 471	83307-84686		
		Conting 463	79826-81577		
	Phytase	Scaffold 8	770352-771503		71
	organic acid	Scaffold 7	186096-186881		85
		Scaffold 6	602817-603782		
Nitrogen metabolims	Urease	Scaffold 6	413884-416278	*B. subtilis* 168	88
	Nitrite reductase	Contig 463	15804-18562		93
	Nitrate reductase	Scaffold 6	355515-361905		96
