# Supplementary material for: Genomic and Experimental Analysis of the Biostimulant and Antagonistic Properties of Phytopathogens of Bacillus safensis and Bacillus siamensis
Source: Microorganisms. 2022 Mar 22;10(4):670. doi: 10.3390/microorganisms10040670 (PMC9024481; doi:10.3390/microorganisms10040670)
Supplement: Supplementary file 1 [file microorganisms-10-00670-s001.zip › microorganisms-1602607 - supplementary/Table S7.pdf]

Table S7. Extracellular enzymes encoded from *B. safensis* RGM 2450 genome.

Type Factor	Factor	Location	Cluster osition	Identity (%)*
Proteases	glutamyl endopeptidase precursor	Scaffold 3	18130-19038	30
	Subtilisin E	Scaffold 3	143446-144597	58
	D-alanyl-D-alanine carboxypeptidase	Scaffold 3	177604-179088	50
	Alkaline serine proteinase	Scaffold 4	89629-91245	53
	Gamma-glutamyltranspeptidase	Scaffold 5	329560-331311	71
	L-alanyl-gamma-D-glutamyl-L-diamino acid endopeptidase	Scaffold 5	359525-360442	49
	Bacillopeptidase F precursor	Scaffold 5	581309-585625	54
	Serine protease AprX	Scaffold 5	829634-830959	65
	Minor extracellular protease Vpr	Scaffold 6	692040-694466	65
	Metalloprotease, putative zinc-binding domain	Scaffold 6	807477-809585	29
	serine alkaline protease	Scaffold 6	904821-905738	38
	prolyl endopeptidase	Scaffold 6	929441-929581	59
Lipase	Lipase precursor	Scaffold 3	946960-947601	74
Plant cell wall-degrading enzymes	Glucuronoarabinoxylan endo-1,4-beta-xylanase	Scaffold 3	103584-104849	88
	Endo-1,4-beta-xylanase	Scaffold 3	200845-201531	50
	Endo-1,4-beta-xylanase	Scaffold 5	725130-726293	NI
	beta-1,4-glucanase (cellulase)	Scaffold 5	720984-722834	NI
	Cellulose 1,4-beta-cellobiosidase (reducing end)	Scaffold 5	722867-724972	NI
	Pectate lyase precursor	Scaffold 6	744308-745336	28
Cell-wall glycopeptides	N-acetylmuramoyl-L-alanine amidase	Scaffold 5	324856-325755	67
	N-acetylmuramoyl-L-alanine amidase	Scaffold 6	12016-12819	54
	Murein hydrolase activator EnvC	Scaffold 6	405898-407160	28
Glucanase	Endo-beta-1,3-1,4 glucanase (licheninase)	Scaffold 4	389496-390224	81
Chitinase	Mannosyl-glycoprotein endo-beta-N-acetylglucosaminidase	Scaffold 6	472794-475424	41
Superoxide dismutase	Superoxide dismutase [Fe]	Scaffold 3	234680-235501	58
	Superoxide dismutase [Mn]	Scaffold 3	563201-563809	86
Lactamase	Beta-lactamase class C-like and penicillin binding proteins	Scaffold 3	648783-649793	27
Ribonucleases	Ribonuclease	Scaffold 6	162246-163124	71
	Ribonuclease	Scaffold 6	349115-349603	64

* Identity of the genes regarding to *Bacillus velezensis* strain FZB42. NI, no identity.
