# Supplementary material for: Genomic and Experimental Analysis of the Biostimulant and Antagonistic Properties of Phytopathogens of Bacillus safensis and Bacillus siamensis
Source: Microorganisms. 2022 Mar 22;10(4):670. doi: 10.3390/microorganisms10040670 (PMC9024481; doi:10.3390/microorganisms10040670)
Supplement: Supplementary file 1 [file microorganisms-10-00670-s001.zip › microorganisms-1602607 - supplementary/Table S8.pdf]

Table S8. Extracellular enzymes encoded from *B. siamensis* RGM 2529 genome.

Type Factor	Factor	Location	Cluster Position	Identity (%)*
Proteases	Minor extracellular protease Vpr	Scaffold 6	286144-288555	98
	Gamma-glutamyltranspeptidase	Scaffold 6	452401-453978	94
	Gamma-glutamyltranspeptidase	Scaffold 8	867610-869385	99
	D-alanyl-D-alanine carboxypeptidase	Scaffold 8	874448-875920	93
	Serine protease AprX	Scaffold 8	1057549-1058877	95
	Subtilisin E	Contig 471	167972-169120	98
	Bacillopeptidase F precursor	Scaffold 8	1323563-1327852	94
	Bacillolysin, extracellular neutral metalloprotease	Scaffold 8	1381280-1382845	97
Lipase	Lipase precursor	Contig 463	72775-73419	97
Plant wall degradation enzymes	Endo-1,4-beta-xylanase	Scaffold 6	417111-417752	94
	Glucuronoarabinoxylan endo-1,4-beta-xylanase	Scaffold 8	976045-977313	94
	Pectate lyase precursor	Scaffold 3	106362-107627	96
Cell-wall glycopeptides	N-acetylmuramoyl-L-alanine amidase	Scaffold 6	995789-996793	73
	N-acetylmuramoyl-L-alanine amidase	Scaffold 7	143741-143881	75
	N-acetylmuramoyl-L-alanine amidase	Scaffold 8	1560706-1561584	96
	N-acetylmuramoyl-L-alanine amidase	Scaffold 9	3579-4762	33
	Alpha-amylase	Contig 463	40924-42903	96
	beta-1,4-glucanase (cellulase)	Scaffold 8	980228-981727	93
	Soluble lytic murein transglycosylase	Contig 471	270137-270823	90
Polisacaride hydrolase	Levansucrase (EC 2.4.1.10)	Scaffold 6	56770-58191	98
	6-phospho-beta-glucosidase (EC 3.2.1.86)	Scaffold 6	183705-183833	100
	Mannosyl-glycoprotein endo-beta-N-acetylglucosaminidase (EC 3.2.1.96)	Scaffold 6	499196-501835	58
	Chitosanase precursor (EC 3.2.1.132)	Scaffold 6	807184-808020	99
	Arabinan endo-1,5-alpha-L-arabinosidase (EC 3.2.1.99)	Scaffold 8	185621-185767	70
	Endo-beta-1,3-1,4 glucanase (licheninase) (EC 3.2.1.73)	Scaffold 6	203049-203780	95
	Arabinoxylan arabinofuranohydrolase (EC 3.2.1.55)	Scaffold 8	974613-97566	91
Ribonuclease	Ribonuclease (Barnase), secreted	Scaffold 6	602222-602671	95
	Extracellular ribonuclease Bsn	Scaffold 6	825907-826773	78
Fe metabolism	Ferredoxin reductase	Scaffold 6	907396-907566	36
	Ferrous iron transport peroxidase EfeB	Scaffold 6	266069-267325	95
Lactamase	Class A beta-lactamase (EC 3.5.2.6)	Contig 471	307694-308608	94
Phytasa	3-phytase (EC 3.1.3.8)	Scaffold 8	770352-771503	95
Oxidative stress	Superoxide dismutase [Mn] (EC 1.15.1.1)	Scaffold 8	462539-463144	99

* Identity of the genes regarding to *Bacillus velezensis* strain FZB42. NI, no identity.
